# The future of embryo engineering and fertility research in interdisciplinary collaboration

**DOI:** 10.3389/fcell.2025.1619050

**Published:** 2025-08-29

**Authors:** Julia Soczyńska, Wiktor Gawełczyk, Julia Papierkowska, Adrian Muzyka, Krzysztof Majcherczyk, Patrycja Obrycka, Mateusz Żołyniak, Sławomir Woźniak

**Affiliations:** ^1^ Department of Human Morphology and Embryology, Student Scientific Society Anatomia-Klinika-Nauka, Division of Anatomy, Wroclaw Medical University, Wroclaw, Poland; ^2^ Student Scientific Group of Heart Diseases, Wroclaw Medical University, Wroclaw, Poland; ^3^ Department of Human Morphology and Embryology, Division of Anatomy, Wroclaw Medical University, Wroclaw, Poland

**Keywords:** embryo engineering, infertility, interdisciplinarity, gene therapy, future development

## Abstract

The increasing prevalence of marital infertility and the persistent desire for offspring have become more significant issues over past decades. Considering the potential genetic, hormonal, and anatomical causes, it is evident that the analysis of infertility is complex, necessitating the development of innovative therapies to address various challenges and dilemmas. The interdisciplinary collaboration of multiple fields fosters scientific progress, such as the development of new research models, reproductive mini-organoids, enhancing the chances of successful parenthood even in challenging cases. Since the fifth decade of the 20th centurymarked by the *in vitro* fertilization of an egg cell, the birth of Louise Brown (the first test-tube baby), the methods of embryo cryopreservation, the discovery of induced pluripotent stem cells (iPSC), and the genetic editing technology CRISPR-Cas9-research has been advancing towards promising directions for studying infertility causes and testing potential therapeutic interventions in controlled conditions. Gene therapy stands as a significant pillar, with 2017 witnessing promising experimental advancements in repairing mutations responsible for hypertrophic cardiomyopathy. Attempts were also made to create Human Immunodeficiency Virus (HIV) immunity by disabling the CCR5 gene, leading to the birth of twins with this variation. Progress in innovative therapies has kept pace with advancements in artificial intelligence, poised to revolutionize reproductive medicine by minimizing human errors. Machine learning (ML) algorithms are being integrated into embryo selection processes, predicting their implantation potential, raising concerns among various nations about eugenics and the interference with human nature. These concerns form a highly debated legal and political pillar. The growing automation is driven by arguments related to the increasing problems of future challenges, such as environmental changes or declining gamete quality. Scenarios under consideration include the development of advanced assisted reproduction technologies and support programs. Theoretical possibilities of alternative methods for organism development are being explored, though they remain constrained by the necessity of rigorous human studies.

## 1 Introduction

The increasing prevalence of infertility has a significant impact on both individual wellbeing and society as a whole. According to recent data, 8%–12% of couples struggle with this issue (Agarwal et al., 2021). In recent years, infertility has gained considerable attention, and the resulting consequences necessitate immediate action, which has led to extensive research in this field (Zarinara et al., 2016). Growing interest in the subject and increasing demand have contributed to the development of therapies and new preventive methods, some of which are directly linked to the hope of eliminating certain diseases and managing their manifestations even before birth. Fertility issues have been recognized since ancient times (Kumar and Singh, 2015). The concept of artificial insemination, which involves the introduction of semen into the female reproductive system, was already documented in antiquity (Sharma et al., 2018). The introduction of genetic engineering in the 1970s (Rahbaran et al., 2021) was perceived as a breakthrough in overcoming reproductive difficulties. Over the past decade, the use of assisted reproductive technologies (ART) in economically developed countries has increased by 5%–10% annually (Benhabbour, 2025). Data indicate that by 2023, nearly 10 million children had been born through *in vitro* fertilization (IVF) (Hariton et al., 2023). However, there remain cases in which science continues to be ineffective (Anwar and Anwar, 2016). Technological advancements in medicine have led to increasingly innovative solutions, which, due to their complexity, often face challenges in clinical implementation—primarily due to rising costs. Despite the existence of numerous modern techniques that appear promising from a scientific standpoint, their practical benefits do not always align proportionally with expectations. This discrepancy represents a significant barrier to further progress in embryonic engineering (Ma et al., 2021). Standard methods, such as embryo transfer—a widely practiced assisted reproduction technique—do not always guarantee success, with an average success rate of approximately 35% (de Santiago and Polanski, 2022), which may be considered a relatively disappointing outcome. These challenges are not the only obstacles in the field. A broader examination of the issue reveals difficulties affecting individuals as well. The literature suggests that infertility in women may be associated with other medical conditions and should raise concerns. According to data, infertile women are more likely to develop endometrial cancer and experience mental health disorders (Hanson et al., 2017). The emotional burden of infertility, which can serve as a precursor to severe health issues, underscores the need for psychological support. Recent studies indicate that psychological interventions play a crucial role in reducing stress among women and increasing pregnancy rates (Rooney and Domar, 2018). The current state of knowledge has been shaped by interdisciplinary influences. The success of assisted reproduction is attributed, among other factors, to extensive collaboration between embryology and endocrinology (Hariton et al., 2023). Furthermore, biotechnology, genetics, and bioinformatics play a crucial role, enabling the analysis of gametes and intervention through pluripotent stem cells (Liu et al., 2022; Wu et al., 2022; Sang et al., 2023). Given the growing and inevitable demand, infertility is now a challenge not only for biotechnology but also for artificial intelligence. Increasingly, AI-based tools are being utilized for data analysis and decision support for both physicians and patients in infertility treatment (Bulletti et al., 2024). Despite the recognized value of interdisciplinary collaboration in infertility research and embryonic engineering, uncertainties remain regarding the long-term consequences and potential effects of complex reproductive technologies. Additionally, further research is essential—not only from a medical perspective but also considering ethical and legal implications. Our focus is on the role of an interdisciplinary approach in understanding infertility, its prevention, and treatment. We believe that a thorough evaluation of the problem—combined with an analysis of existing methods, technologies, and research outcomes—will help define the future direction of embryonic engineering and fertility studies. We emphasize the necessity of extensive research through interdisciplinary cooperation and highlight the potential long-term benefits of such collaboration, which may ultimately improve public access to medical knowledge and services. This, in turn, serves as a strong altruistic motivation to advance scientific research and continue progress in the field.

**TABLE 1:** Summary of studies.

	Study	Year	Methods	n	Aim	Outcomes
1	Lee et al.	2018	CRISPR-based gene editing, done by CRISPR-Gold delivered by intracranial injection	total of 11 mice (6- control, 5-for CRISPR-Gold)	CRISPR-Gold is to deliver Cas9 and Cpf1 into the brain, and then lower activity of mGluR5, therefore treating fragile X syndrome	CRISPR-Gold treated neurons did not show any adverse effect caused by the treatment. Behavioural changes has been observed that led to the conclusion that this method may be able to treat neurological diseases
2	Raposo	2019	CRISPR-based gene editing, done by CRISPR-Cas9	2 (twins)	To immunize twins from HIV by utilizing CRISPR-Cas9 technique that modify the CCR5 gene	Still to be fully delivered, but it is belived that the trial was a success
3	Briski et al.	2024	CRISPR-based gene editing done by CRISPR-Cas9	Not applicable	To modify the embryonic pigs genome in order to make pigs tissues less likely to be rejected by a human host after transplantation	CRISPR-Cas9 technique surpasses traditionally obtained tissues through *in vitro* methods or ICSI
4	El Hachem et al.	2014	Cryotechnology is employed to freeze oocyte	1	Cryopreserved oocytes were used for fertilization and pregnancy	Both pregnancy and childbirth was successful
5	Vuong et al.	2020	One group was randomized to undergo standard oocyte maturation and the other was to undergo capacitation *in vitro* maturation	80 (half into each group)	To evaluate which technique is safer and superior	Use of capacitation *in vitro* maturation system was associated with better results such as improved maturation and clinical pregnancy rates than standard oocyte maturation
6	Donnez et al.	2004	Cryotechnology is employed to freeze a fragment of ovarian tissue	1	Cryopreserved ovarian tissue is used for treating ovarian failure due to cancer treatment	Ovary regained its functions, enabling the patient to conceive naturally, even after chemotherapy
7	Long et al.	2024	Sperm was collected from infertile men, then intracytoplasmic sperm injection was administered	7	To enable couples in which men suffer from primary infertility to have children	Three out of seven couples have given birth to five healthy babies
8	Wang et al.	2018	516 oocytes were fertilized by intracytoplasmic sperm injection	516 (286 – fresh embryos, 230 – frozen-thawed embryos)	To evaluate which source of an embryo is superior	Frozen-thawed embryos showed less complications with the pregnancy, but also the rate of miscarriage was significantly less than the other group

## 2 The role embryology in understanding the causes of infertility

Causes of infertility may stem from genetic, hormonal, or anatomical factors affecting gametogenesis, fertilization, or early embryo development. Oxidative stress is considered the most significant pathological factor in infertile men. Studies have shown that an increase in reactive oxygen species (ROS) can lead to substantial disruptions in the redox balance within sperm cells. Oocyte maturation arrest is a key cause of female infertility linked to PABPC1L function. Targeting MOS overexpression may rescue affected oocytes, and *in vitro* gametes from stem cells offer future therapeutic potential (Wang et al., 2023). Reproductive mini-organoids, introduced as novel research models, enable the *in vitro* study of cellular and molecular processes. They show great promise and provide an ideal platform for investigating the causes of infertility and testing potential therapeutic interventions under controlled conditions.

**FIGURE 1:**
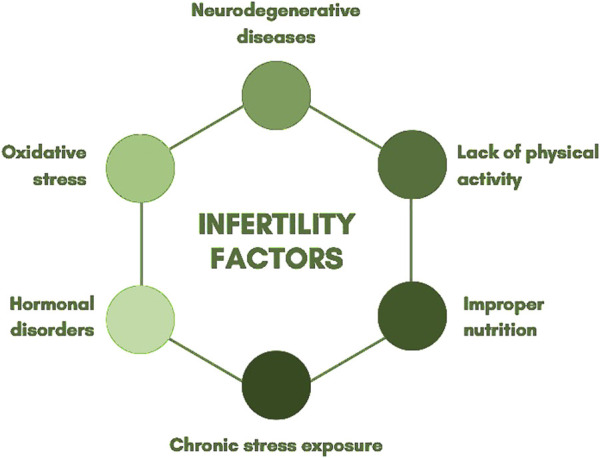
Infertility factors.

Infertility is clinically defined as a disorder of the reproductive system characterized by the inability to achieve pregnancy after 12 months or more of regular, unprotected sexual intercourse (Makar and Toth, 2002). Research indicates that male factors contribute to 30%–50% of infertility cases (Eisenberg et al., 2023). The factors contributing to infertility are highly complex. Among them are physical inactivity, poor nutrition, chronic stress exposure, and excessive physical and psychological strain. These factors can lead to hormonal imbalances in both sexes, resulting in anovulatory cycles in women and decreased semen quality in men (Głowacka, 2018). Oxidative stress is a contributing factor not only to infertility but also to neurodegenerative diseases (such as Alzheimer’s and Parkinson’s disease) and cancer (Grosicka-Maciag, 2011). A low concentration of ROS is an essential component for the proper functioning of male reproductive cells. ROS play a crucial role in DNA condensation and regulate apoptosis and proliferation, which are key mechanisms in controlling spermatogenesis productivity (Tafuri et al., 2015). The human body produces antioxidant enzymes, such as glutathione peroxidase (GPx), superoxide dismutase (SOD), and catalase, which neutralize ROS and protect cells from oxidative stress. SOD catalyzes the conversion of superoxide radicals into hydrogen peroxide, which is subsequently broken down by GPx and catalase into water and oxygen, preventing the accumulation of toxic byproducts. The proper functioning of these mechanisms is crucial for maintaining the structural and functional integrity of sperm cells. Their impairment can lead to reduced motility and, consequently, decreased fertilization capability and severe DNA damage (Netherton et al., 2020; Vozdova et al., 2022). Elevated ROS levels are among the most significant endogenous factors causing DNA damage (Xu et al., 2024). They negatively impact sperm motility and further reduce ATP levels (Thomas et al., 1997). Due to the condensed chromatin structure and limited availability of repair mechanisms, the repair of single- and double-strand breaks is highly restricted. Oxidative stress induces sperm DNA fragmentation, reducing genetic integrity and increasing the risk of miscarriages, implantation failure, and the transmission of genetic defects to offspring (Wang et al., 2025). ROS generated during oxidative stress include malondialdehyde (MDA), protein carbonyl (PC), and glutathione disulfide (GSSG), which have detrimental effects on the body (Valko et al., 2007). The sperm plasma membrane differs from that of somatic cells due to its specific functions. Its susceptibility to oxidative stress results from the high content of unsaturated fatty acids, particularly docosahexaenoic acid (DHA) and polyunsaturated fatty acids (PUFAs), which play a critical role in human sperm function (Henkel, 2010). Due to their unique structure and function, sperm cells are particularly vulnerable to ROS. They have low levels of reduced glutathione and minimal antioxidant enzyme activity, limiting their ability to neutralize ROS. Unlike other cells, spermatozoa possess impaired mechanisms for repairing DNA damage (Bauché et al., 1994; Aitken, 1995). Studies indicate that increased ROS concentrations can cause oxidative-reductive imbalance in sperm, leading to structural damage and potential pathological alterations (Walczak-Jędrzejowska, 2015). Disruptions in spermatogenesis can lead to premature apoptosis of sperm cells, adversely affecting sperm count and quality (Grosicka-Maciag, 2011). ROS can cause DNA strand breaks, leading to base loss or modifications, such as the formation of 8-oxo-G lesions. Sperm DNA damage is positively correlated with lower fertilization rates in IVF, impaired implantation success, increased miscarriage rates, and a higher incidence of childhood diseases, including cancer (Lewis et al., 2008). In ART, the presence of fragmented DNA further hampers successful fertilization and increases the risk of embryonic developmental disorders (Wang et al., 2025). Oxidative stress arises due to an imbalance between ROS production and the follicular fluid’s capacity to detoxify these species in the environment surrounding the developing oocyte in the ovary. This condition is associated with reduced oocyte quality and lower fertilization rates, potentially leading to decreased pregnancy success rates (Zaha et al., 2023). Redox signaling can be utilized in male infertility therapy to develop new diagnostic tools. ROS, such as superoxide anion, hydrogen peroxide, nitric oxide, and peroxynitrite, regulate redox signaling during sperm capacitation by activating protein kinases and inhibiting protein phosphatases, leading to specific phosphorylation modifications (O’Flaherty, 2025). A key role in this process is played by peroxiredoxin 6 (PRDX6), which possesses both peroxidase and calcium-independent phospholipase A2 (iPLA2) activity. Additionally, PRDX6 regulates the lysophosphatidic acid signaling pathway, which is essential for maintaining sperm viability (Ryu et al., 2017). Models that mimic tissues and organs, developed through advances in organoid research, currently serve as a bridge between *in vitro* and *in vivo* studies. Organoids replicate numerous biological and pathological characteristics of organs, including organ-specific functions and the presence of multiple specialized cell types (Lancaster and Knoblich, 2014). Organoids exhibit the features of various reproductive organs such as the uterus, ovaries, fallopian tubes, and even trophoblasts. They present an attractive alternative to animal and conventional *in vitro* models due to their genetic stability and prolonged adherence to the tissue of origin during extended cultures (Heidari-Khoei et al., 2020). Research on *in vitro* pregnancy modeling has focused on the use of trophoblasts. However, the results of primary trophoblast isolation and culture have been insufficient for their application in organoids. This limitation arises because trophoblasts are highly mature cells that neither differentiate nor proliferate efficiently *in vitro*. Even when successfully isolated, they tend to fuse, forming multinucleated cells (Park et al., 2023). Male organoids derived from human pluripotent stem cells (hPSCs) follow the developmental pathway of the embryonic gonad, differentiating into multipotent progenitors and subsequently specializing into testicular support cells and interstitial cells. Studies have confirmed the activity of these generated cell types through marker expression analysis, demonstrating the architectural organization of tissues (Pryzhkova et al., 2022). A significant breakthrough was the discovery of persisting ovarian stem cells (OSCs) in adult ovaries, which enabled the development of an alternative approach to the rare OSCs pool with the aim of obtaining fertilization-capable oocytes. Oocyte growth and maturation were achieved through supplementation with estrogen receptor antagonists, bone morphogenetic protein 15 (BMP15), follicle-stimulating hormone (FSH), growth differentiation factor 9 (GDF9), fibroblast growth factor (FGF), epidermal growth factor (EGF), and human chorionic gonadotropin (hCG) (Haider and Beristain, 2023). Studies show that dark colour of the cytoplasm, homogeneous cytoplasmic granularity, refractile bodies, or a fragmented first polar body do not affect the process of diagnosis and treatment. Negative impact on the treatment process infertility treatment has been indicated to presence of cytoplasmic vacuoles clusters of smooth endoplasmic reticulum and centrally located cytoplasmic granularity (Nikiforov et al., 2022). One of the most important causes of female infertility is oocyte maturation arrest. PABPC1L, the predominant poly(A) binding protein in *Xenopus*, mouse and human oocytes, plays an important role in the translational activation of maternal mRNAs (Wang et al., 2023). Studies which implicating MOS overexpression in PABPC1L variant oocytes are a good direction for therapeutic progress. The oocytes or early embryos from a woman bearing pathogenic PABPC1L variants might be “rescued” by introducing MOS siRNA or MAPK inhibitors (Ding and Schimenti, 2023). In the future, unlimited gametes developed *in vitro* from induced pluripotent stem cells generated from parental skin cells may replace the low number of natural oocytes maturing after hormonal stimulation (Murakami et al., 2023). An overview of the relevant elements is presented in Figure 1.

## 3 History

**FIGURE 2:**
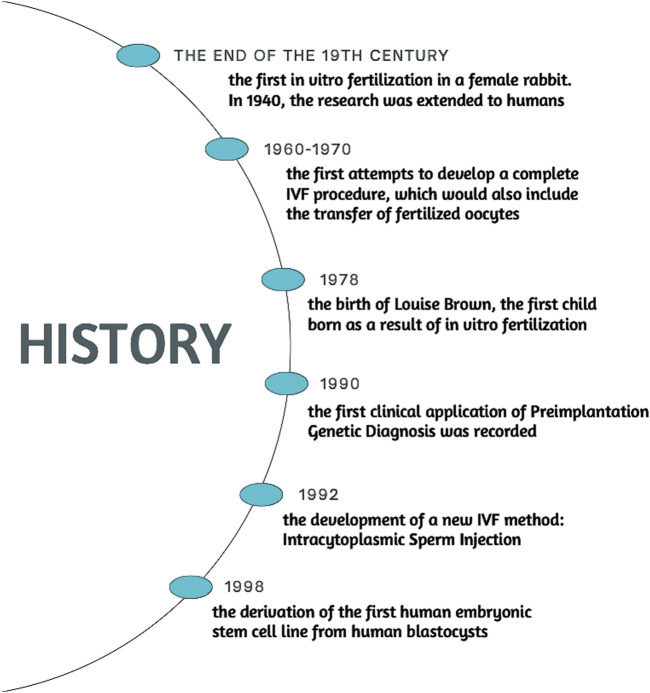
History.

The history of fertility research dates to XVII century, when Antonie van Leeuwenhoek for the first time in history observed human spermatozoa under microscope. Another pioneer in discovering fundaments of embryology was Karl Ernst Von Baer, who first described dog oocyte and its role in reproduction (Heape et al., 1981). In the XIX century, researchers started to put interest in manipulating the embryos. In 1890 a first successful transfer of mammalian embryo was conducted. Walter Heape managed to carry out an embryo transfer between two rabbits does (Heape, 1891; Biggers, 1991). This experiment created a foundation for initial experiments in IVF. Animal research was conducted, one of the most notable being Pincus and Enzmann achieving the first fertilization outside of the body of a female rabbit. This concept was picked up in 1940s by Miriam Menkin, who then extended the research to human subjects. In collaboration with John Rock, they collected human oocytes from women scheduled for hysterectomy. The result of the research was Menkin performing the very first human egg fertilization outside the body (Menkin and Rock, 1948). Despite the success in fertilizing the oocyte, the zygote was not transferred into uterus. In 1960s–1970s Edwards and Steptoe undertook the issue with the aim of developing a complete IVF procedure that would also involve the transfer of fertilized eggs, leading to successful pregnancy. Back then, egg collection for IVF posed a significant challenge, for accessing woman’s ovaries was highly invasive and took a considerable amount of risk. Steptoe introduced a new method of retrieving oocytes via laparoscopic needle aspiration. Having collected the oocytes, they fertilized the eggs with sperm cells and cultured them on a special medium. Methods Edwards and Steptoe used, made it possible to extend the embryo culture to the blastocyst stage, while prior to their work embryos could only last for 2–3 days. Longer culture period had its advantage, as it allowed researchers to select most viable embryos. They developed microscopic methods for tracking embryonic cell division, assessing the cleavage rate and evaluating the quality of the embryo based on parameters such as number of cells, evenness of the cells and lack of fragmentation (Steptoe, 1978; Edwards et al., 1980; Edwards et al., 1981). In 1978, after combining all the advances in fertility research, Edwards and Steptoe’s work culminated in birth of Louise Brown, the first baby born with the *in vitro* fertilization method (Kamel, 2013). Such achievement has opened a new era of assisted reproduction technology as well as for whole branch of modern embryonal engineering. Only 6 years later in the Netherlands, twins were born following embryo cryopreservation (Zeilmaker et al., 1984), which had a massive impact on reproduction, as later fertility would be able to be preserved as one struggled with conditions detrimental to possibility of pregnancy, such as ovarian failure by high estrogen levels in breast carcinoma patients (Oktay et al., 2003; Baynosa et al., 2009). In 1990, first clinical use of Preimplantation Genetic Diagnosis was reported. Team at Hammersmith Hospital in London utilised polymerase chain reaction, to analyse the embryonal DNA for X-linked diseases (Handyside et al., 1990). Two years later, they were able to implement preimplantation testing for cystic fibrosis (Handyside, 1992). The same year brought another milestone invention in IVF procedure. Gianpiero D. Palermo and his colleagues from Vrije Universiteit in Brussels observed that the sperm they were using had difficulties fertilizing the oocyte in a traditional method, which back then was culturing egg cell with sperm on a Petri dish. This led the team of scientists to attempt to inject a single sperm cell inside the ovum, which happened to be successful, resulting in four live births (Palermo et al., 1992). A new method of Intracytoplasmic Sperm Injection (ICSI) was developed and to this day it is a procedure of choice in cases of IVF, where the man has low sperm count, severely impaired motility or abnormal morphology (Chen et al., 2022). In 1998, James Thomson derived the first embryonic stem cell lines from human blastocysts (Thomson et al., 1998; Yu and Thomson, 2008). This was a critical breakthrough, fundamentally changing the landscape of biomedical research, regenerative medicine and genetics. Thomson’s work demonstrated that human embryonic stem cells (hESCs) could be cultured for an indefinite period in the lab without losing their pluripotency, allowing for long-term cell studies. As to create stem cells, human embryo must be destroyed, Thomson’s discovery has triggered numerous ethical debates, whether it is moral to manipulate embryos, which by some are morally considered to be equivalent to an adult human being (Juengst and Fossel, 2000; De Wert and Mummery, 2003; de Miguel-Beriain, 2015). An alternative to hESCs was invented in 2006 by Shinya Yamanaka of Kyoto University. He and his team discovered that four transcription factors: Oct4, Sox2, Klf4, and c-Myc are the key ones for pluripotency at early embryonic cells. Using these factors, Yamanaka’s scientists team managed to reprogram adult mouse fibroblasts into iPSC (Takahashi and Yamanaka, 2006). Only a year later, the same reprogramming was applied to human fibroblasts, successfully creating human iPSCs. Stem cells obtained this way are considered to be an ethically acceptable source of pluripotent cells, as their acquisition do not require destruction a human embryo (Takahashi et al., 2007). To this day, iPSCs are used in variety of instances, for example disease modelling or regenerative medicine. Clinical trials for using iPSCs in regenerative therapies have been initiated, with promising results in areas such as retinal degeneration and neurodegenerative diseases (Mandai et al., 2017; Beghini et al., 2024). The methods mentioned above are only the milestones of reproductive medicine and embryonal engineering. From the birth of Louise Brown in 1978, those branches of science have exploded with new concepts coming year after year and brand new innovations keep coming nowadays with increasing tempo. Figure 2 outlines the chronological development of the issue.

## 4 From fertilization to embryo screening: ICSI and PGT in contemporary IVF practice

According to a WHO report from 2023, it is estimated that approximately 17,5% of the adult population worldwide experience infertility [1 in 6 people]. IVF, while still triggering a considerable dose of controversy, has already become a standard procedure for couples unable to conceive naturally, as well as a core procedure in today’s assisted reproductive medicine. Since the birth of Louise Brown in 1978, it has been used for decades with a view to treat a whole range of infertility issues, including male infertility, fallopian tube occlusion, ovulatory disorders like polycystic ovary syndrome (PCOS) as well as idiopathic infertility (Elder, 2001; Niederberger et al., 2018). According to a 2019 report by European Society of Human Reproduction and Embryology (ESHRE), 1 077 813 ART cycles were reported from 40 countries with an estimated rate of 1581 cycles per million inhabitants (Smeenk et al., 2023). In 2019 the number of total cycles increased to 784192, with a constantly increasing trend (Wyns et al., 2022). The IVF procedure routinely incorporates techniques related to embryo handling and genetic testing, as they are used throughout the process–from gamete collection and fertilization to embryo assessment, selection, and uterine transfer.

ICSI is a widely used method in IVF procedure, predominantly used in male factor infertility including cases with low sperm count, poor sperm motility, abnormal sperm morphology or high DNA fragmentation (Hamberger et al., 1998; Van Steirteghem et al., 2002; Haddad et al., 2021). ICSI is also indicated after two or more conventional IVF attempts. Moreover, ICSI is also necessary if sperm is obtained through surgical methods like testicular sperm extraction (TESE) or epididymal aspiration (Hamberger et al., 1998; Esteves et al., 2015). ICSI is sometimes used in cases of unexplained infertility or low ovarian response to stimulation (Haddad et al., 2021). As of today, ICSI effectiveness has been proven to be higher for severe male factor infertility. In cases, where males present with normal sperm parameters or mild infertility, studies do not show clear benefit over classic IVF procedure in terms of live birth rates, clinical pregnancy rates or miscarriage rates (Esteves et al., 2018; Cutting et al., 2023). Despite being used in non-male infertility factors, it is a subject of controversy, as studies do not indicate explicitly that ICSI brings better results in such cases. A meta-analysis by Ting Geng et al. did not find any clear benefit over conventional IVF for couples with non-male factor infertility. Numerous meta-analysis studies, including European multicenter analysis indicated that while ICSI may have a slightly higher fertilization rate, it does not translate into better clinical outcomes such as pregnancy or live birth rates (Li et al., 2018; Drakopoulos et al., 2019; Geng et al., 2020). ICSI, however, may be associated with a higher risk of perinatal outcomes, which should be considered when choosing this method (Li et al., 2018). Children conceived through ICSI may have an increased risk of epigenetic disorders congenital malformations and chromosomal abnormalities compared to naturally conceived children (Sciorio and Esteves, 2022). A meta-analysis by Zan Zheng et al. reported higher relative risk of chromosomal defects, urogenital (notably hypospadias) and circulatory system malformations with 1.36; 1.18 and 1.22 RR value accordingly. The same study points out, though, that other types, such as cleft lip/palate, musculoskeletal, nervous, and digestive system malformations, do not show significant differences between ICSI and natural conception (Zheng et al., 2018). The potential cardiovascular risk is also supported by another meta-analysis by Xiao-Yan et al., as it states that children and young adults conceived via ICSI show higher systolic and diastolic pressure on average in comparison to naturally conceived peers. While the increase in blood pressure is minor (SBP ∼2–5 mmHg higher and DBP ∼1–3 mmHg higher), it remains statistically significant, yet its impact is still a question to be answered (Guo et al., 2017). Contrary to studies presenting increased risk, some of the large studies and meta-analyses show no significant increase in *de novo* chromosomal abnormalities in ICSI offspring compared to natural conception, though some unadjusted data suggest a small increase; overall, the absolute risk remains low (Berntsen et al., 2021; Yuan et al., 2022). Interestingly, the increased risk of congenital malformations is more pronounced in multiple pregnancies. When analyses are restricted to singletons, the difference is smaller (Wennerholm et al., 2000; Chen et al., 2018). Many chromosomal abnormalities and some malformations are linked to underlying parental genetic factors, especially male infertility, rather than the ICSI procedure itself (Simpson and Lamb, 2001; Van Steirteghem et al., 2002). The question of ICSI’s epigenetic impact is still a subject of debate. Literature reviews and observational studies show that both ICSI and IVF can alter methylation at imprinted gene regions, which are critical for normal development. These changes may be linked to the increased risk of certain imprinting disorders in ART-conceived children (Sciorio and Esteves, 2022). Rare cases of imprinting disorders, such as Angelman syndrome, have been reported in ICSI-conceived children, possibly due to interference with the establishment of maternal imprints during early development (Cox et al., 2002). While ICSI is associated with small but significant differences in DNA methylation at thousands of CpG sites in cord blood compared to natural conception, these changes generally remain within the normal range of variation and do not unanimously translate to adverse clinical outcomes (Estill et al., 2016; El Hajj et al., 2017). Several meta-analyses also indicated higher risk of obstetric outcomes in singleton pregnancies resulting from IVF/ICSI, including both spontaneous and iatrogenic preterm birth below 37 weeks. The odds ratio vary from 1.7 to even 2 times higher than natural conception, depending on the study (Pandey et al., 2012; Cavoretto et al., 2018; Cavoretto et al., 2020; Salmeri et al., 2024).

Prior to the implantation, embryos are tested for potential disorders, that might have resulted from previous steps, as well as from genetic factors such as parents having positive family history towards genetic disorders. Procedures of testing the embryos for genetic disorders widely go by the name of Preimplantation Genetic Testing (PGT). It covers two stages: cell biopsy and then subjecting it to genetic analysis. Currently, three main methods of collecting the testing material are conducted: blastomere biopsy, trophectoderm biopsy (TB) and polar body analysis (Aoyama and Kato, 2020; de Rycke et al., 2020; Greco et al., 2020). The first one is performed on day 3 of the embryonal development, as 6-8 cell stage is reached. The zona pellucida is opened using either mechanical drilling, acid Tyrode’s solution or laser-assisted hatching (LAH), the last of which being the method of preference in modern clinical practice. The studies do not unanimously indicate domination of a certain method, while some of the papers’ results are in favour of LAH. A study by Gabrielsen et al. points out that laser drilling resulted in more intact blastomeres than acid Tyrode (98.3% vs. 95.2%, p = 0.02), while not having statistically significant differences in implantation rates (Joris et al., 2003). Contrary to that, some studies show that LAH method results in higher implantation rates as well as precision and consistency compared to chemical or mechanical techniques (Balaban et al., 2002; Makrakis et al., 2006; Nishio et al., 2006). After opening of the zona pellucida, one or two blastomeres are extracted for genetic analysis. Studies show, however, that blastomere biopsy might affect viability and potential of development of the embryo, leading to mosaicism (Cimadomo et al., 2016). Another method is TB, which is conducted on day 5 or 6, during the blastocyst stage. Cells from the trophectoderm layer are removed for genetic analysis, while leaving the inner cell mass (ICM) intact (De Vos and De Munck, 2025). The method is reported to provide a more comprehensive genetic assessment, reducing the risk of mosaicism (Coll et al., 2018). Compared to earlier-stage biopsies, TE biopsy is reported to have a minimal detrimental effect on implantation potential, as well as relatively high live birth rates; thus, having significant clinical utility (Cimadomo et al., 2016). A meta-analysis by Mao et al. indicated that while there might be a moderate increase in the risk of preterm delivery, TE biopsy for PGT does not affect other obstetric and neonatal outcomes in comparison with standard IVF (Mao et al., 2024), therefore making it a standard method of biopsy today (Coll et al., 2018). Last mentionable method is polar body (PB) analysis. The first and/or second polar body is aspirated via a micropipette and then transferred into a tube for genetic analysis. This method is primarily used for aneuploidy screening and monogenic disorder detection especially in cases where embryo biopsy is not preferred for legal, ethical, religious or technical reasons. PB analysis is efficient for detecting single-gene disorders with a study by Kuliev and Rechitsky reporting an accuracy rate exceeding 99% for PB-based PGT (Kuliev and Rechitsky, 2011). PB analysis has its use especially concerning meiotic abnormalities of maternal origin, which, according to a randomized clinical trial by Verpoest et al., account for approximately 90% of oocyte meiotic errors (Verpoest et al., 2018). According to a review study, PB analysis has an aneuploidy detection rate of 67% (Van Der Ven et al., 2008). While effective for errors of maternal origin, this type of analysis has its limitation, as it does not include paternal genetic contributions; therefore, not fully predicting the resulting ploidy status of the embryo after fertilization (Salvaggio et al., 2014). PGT has been found to have several clinical implications while selecting embryos for an IVF procedure. It can be used to test embryos for aneuploidy, monogenic disorders, structural rearrangements or polygenic risk, with testing methods being called accordingly: PGT-A, PGT-M, PGT-SR, PGT-P. PGT-A is often used for screening for numerical chromosomal abnormalities, which are a leading cause of implantation failure and miscarriage. Despite some debate over its efficiency, it is applied in clinical settings, especially among patients with repeated IVF failures or advanced maternal age (Aydin et al., 2019; Fesahat et al., 2020; Campbell-Forde et al., 2024). PGT-M on the other hand, is particularly beneficial tool for couples at risk of passing monogenic disorders to their offspring. PGT-M has been successfully applied in genetic conditions such as beta thalassemia, cystic fibrosis and muscular dystrophies like Duchenne Muscular Dystrophy (Aktuna et al., 2019; Mamas et al., 2022). PGT-M has also been found to be able to detect rare conditions such as early infantile encephalopathy 5 (EIEE5), xeroderma pigmentosum, congenital merosin-deficient muscular dystrophy and phenylketonuria (Aktuna et al., 2019; Prokhorovich et al., 2019). Interestingly, PGT-M can also be used for screening genetic predisposition to cancer, particularly BRCA1/2 mutations (Madjunkova et al., 2024). PGT-SR is a technique used to identify and select embryos with balanced chromosomal structures, which is particularly helpful for couples with known chromosomal rearrangements (Christianti and Legiran, 2024). Studies have shown a live birth rate of 66.6% per embryo transfer in couples using PGT-SR (Shetty et al., 2022). As far as efficiency of PGT is concerned, PGT-A has been shown to improve implantation rates and reduce miscarriage rates. However, its impact on live birth rates in general population remains less clear with some studies indicating no significant improvement. Due to potential misdiagnosis or biopsy-related damage it might lead to embryo wastage (Pagliardini et al., 2020; Simopoulou et al., 2021). Additionally, the efficiency is negatively impacted when using frozen-thawed oocytes (Martino et al., 2024). Studies have shown that embryo selection and clinical outcomes can be enhanced, if PGT-A is used alongside PGT-M and PGT-SR. Results show higher pregnancy and live birth rates, as well as lower miscarriage rates (Campbell-Forde et al., 2024; Madjunkova et al., 2024).

Apart from combining different methods of PGT, introduction of new molecular diagnostic methods had an important influence in efficiency of embryo testing. Primary methods of testing classically included fluorescent *in-situ* hybridization (FISH), array comparative genomic hybridization (aCGH), quantitative polymerase chain reaction (qPCR), single nucleotide polymorphism microarray (aSNP). FISH was widely used for detection of chromosomal abnormalities and was a method of preference for PGT, especially for identification of aneuploidies and gender selection (Piyamongkol, 2020; Takeuchi, 2021; Moustakli et al., 2024). However, it had its limitations in detecting complex genetic issues and mosaicism (Gontar et al., 2019; Moustakli et al., 2024). aCGH was then introduced as a more sophisticated method, being able to provide detailed copy number variations across all chromosomes, allowing for more complete chromosome screening. It was used for both aneuploidy and segmental rearrangement testing. While offering a broader analysis compared to FISH, it was eventually replaced by next-generation sequencing, which revolutionized PGT (Sekhon et al., 2017). Next-generation sequencing (NGS) technologies operate on principle of massively parallel sequencing, where spatially separated, clonally amplified DNA templates or single DNA molecules are sequenced in a flow cell. The process involves iterative cycles of polymerase-mediated nucleotide extensions or oligonucleotide ligations, producing sequence outputs ranging from hundreds of megabases to gigabases (Voelkerding et al., 2009; Wang, 2021). Study by W. Niu et al. showed that NGS-based PGT-A resulted in higher clinical pregnancy rates, lower miscarriage rates and higher healthy baby rates in comparison with SNP array-based methods. NGS offers higher resolution and broader diagnostic capability, as unlike traditional methods, NGS can simultaneously assess aneuploidies, monogenic disorders and structural rearrangements from a single biopsy. What is more, NGS can be more effective when it comes to expenses. With vast automation and the ability to process multiple samples simultaneously, results are provided quickly and the whole workflow is significantly enhanced (Tan et al., 2023). Studies have also presented high concordance rates between NGS results and initial diagnoses; thus, the technique provides considerable reliability and accuracy (García-Pascual et al., 2020).

While offering potential benefits, PGT also carries certain dose of risk. A large cohort study found that pregnancies achieved with PGT (specifically using trophectoderm biopsy) had a significantly higher risk of preeclampsia compared to IVF pregnancies without PGT (10.5% vs. 4.1%; aOR = 3.02). This increased risk remained even when only singleton pregnancies were analyzed (Zhang et al., 2019). A systematic review and meta-analysis also reported that PGT pregnancies have a higher risk of hypertensive disorders of pregnancy, including preeclampsia, compared to spontaneously conceived pregnancies (RR = 3.12) (Zheng et al., 2021). In opposition, a large multicenter retrospective cohort study by Cozzolino et al. found that, after adjusting for confounding factors, PGT-A was not associated with an increased rate of preeclampsia in singleton pregnancies compared to IVF/ICSI without PGT (Cozzolino et al., 2023). There is also a noted increase in risk of placenta previa in PGT pregnancies, although the evidence is not consistent across all studies (Zhang et al., 2019; Hou et al., 2021). It has been observed by some studies that PGT pregnancies have a higher risk of preterm delivery and low birth weight compared to spontaneously conceived pregnancies, though these risks remain lower in comparison to other IVF/ICSI pregnancies (Hou et al., 2021; Zheng et al., 2021). Overall PGT does not significantly increase risks of adverse obstetric and neonatal outcomes in comparison to IVF/ICSI pregnancies, though hypertensive disorder risks should be considered, as they remain higher (Cozzolino et al., 2023).

## 5 Cutting-edge therapies and the evolution of reproductive technologies

The advancement of technology and progress in innovative therapies are revolutionizing reproductive medicine. Significant developments, particularly in the field of artificial intelligence (AI), are being observed. AI-based tools enhance the accuracy of semen analysis by automating the assessment of sperm morphology and motility, thereby minimizing human error. Similarly, ML algorithms contribute to improving IVF by optimizing embryo selection processes. The integration of informatics and big data analysis enables the personalization of treatment, optimizing decision-making and paving the way for precision medicine in reproductive care. Over the past decades, the potential of AI in medicine has been widely theorized. However, only recently have physicians and computer science specialists begun to uncover its real-world clinical applications, driven by recent technological advancements (Chu et al., 2019). Data provided by European countries for the studies, include treatments with IVF, ICSI, IVM, frozen oocyte replacement (FOR), IUI with husband’s/partner’s semen (IUI-H), and with do-nor semen (IUI-D), preimplantation genetic testing (PGT; pooled data), frozen embryo transfer (FET), egg donation (ED) (Smeenk et al., 2023). Research findings indicate that phthalic acid (PA) and its isomers exhibit toxic properties toward the reproductive system both *in vitro* and *in vivo*. They particularly affect sperm motility and induce cytotoxicity in testicular cells. Among the analyzed isomers, PA demonstrated the highest toxicity, suggesting its potential use as a surrogate biomarker for reproductive toxicity in cases of exposure to phthalate mixtures (Kwack and Lee, 2015). AI has the capability to analyze vast amounts of data, particularly video and images, making it especially useful in the assessment and selection of gametes and embryos. Various AI models have been developed for this purpose, some of which have demonstrated high efficiency (Si et al., 2023). Among the widely used and well-performing AI models are ML algorithms, including decision trees (DT), support vector machines (SVM), and neural networks (Kohli et al., 2017). ML utilizes computer programs to learn from training datasets and to generate predictions within the scope of a predefined task. By providing the computer program with datasets and desired outcomes, ML algorithms are developed. This enables the prediction of future outcomes for specific tasks. Due to its ability to handle large volumes of complex medical data—an area where traditional algorithms often struggle—this technique has achieved significant success in the field of medicine (Lustgarten Guahmich et al., 2023).

In ML based on neural networks, algorithms are created in which machines learn and solve problems in a manner similar to the human mind (Iqbal et al., 2021). Imaging was obtained using three fundamental techniques: cone-beam computed tomography (CBCT), digital imaging of embryos after egg release, and magnetic resonance imaging (MRI) (Lasota, 2023).

Research demonstrates that a neural network used for fertility data analysis can predict Sperm Penetration Assay (SPA) and Penetrak test outcomes based on semen analysis. The neural network outperformed traditional statistical methods (LDFA and QDFA), accurately predicting Penetrak test results in over 80% of cases and SPA test results in nearly 70% (Lamb and Niederberger, 1993).

The causes of infertility may stem from one or both partners, and in some cases, the cause remains unknown due to its often multifactorial nature. The complexity of treatment arises, among other factors, from the influence of numerous variables on gamete quality, embryo development, and the embryo’s ability to implant (Cybulska, 2019). Clinical data, as well as microscopic-level visualizations, undergo objective analysis through the application of artificial intelligence algorithms in IVF laboratories (Jiang and Bormann, 2023). To maximize pregnancy rates and optimize IVF procedures, a precise assessment of embryo viability is essential (Saeedi et al., 2017). AI algorithms, with their ability to analyze and synthesize large datasets, represent a promising tool for assessing sperm quality, thereby enhancing the objectivity and precision of analytical methods (Panner Selvam et al., 2024). The combination of AI with automated analysis of embryos and blastocysts is highly promising (Filho et al., 2012). According to studies, the use of the SVM method with a polynomial kernel can achieve an accuracy of approximately 95%. Based on the results, it can be concluded that SVM demonstrates greater stability and effectiveness in analyzing small datasets (Septiningrum et al., 2022). Computer-assisted sperm analysis systems (CASA) play a significant role in semen evaluation (Goodson et al., 2017). To obtain more objective and precise results, the development of automated image-based methods is essential. Moreover, research indicates that up to one-third of male infertility cases are idiopathic (Gudeloglu et al., 2014). Successful fertilization requires proper sperm motility. Highly decorated doublet microtubules (DMTs) form the backbone of the sperm tail, which is responsible for motility. The use of cryo-electron microscopy (cryo-EM) and AI has enabled the determination of the DMT structures of mouse and human sperm. Additionally, an atomic model of a 48-nm DMT repeat unit in mouse sperm has been developed (Zhou et al., 2023). AI has played a significant role in the analysis of sperm morphology and motility, as well as in ART, aiming to select the most suitable sperm for reproduction (Wang et al., 2019). In reproductive medicine, AI research primarily focuses on image-based analysis of sperm cells and embryos, as well as on predicting ART outcomes. In some medical areas, AI has demonstrated effectiveness comparable to or even surpassing that of clinical specialists (Filho et al., 2012), which raises concerns about the potential replacement of professionals. However, AI should be viewed as a tool that enhances the work of clinicians (Kohli et al., 2017). Surgical sperm retrieval in men with non-obstructive azoospermia (NOA) enables the isolation of sperm from testicular biopsies for use in ART procedures, such as IVF or ICSI. Thanks to advances in AI, there is hope for the development of tools that enable the identification of sperm within testicular tissue (Bachelot et al., 2023). Treatment for patients with NOA often involves a procedure known as microdissection testicular sperm extraction (m-TESE), which has demonstrated a high sperm retrieval success rate of up to 64% (Glina and Vieira, 2013). In men with NOA undergoing TESE, successful sperm retrieval can be predicted using ML algorithms, with promising outcomes (Qi et al., 2021). Research emphasizes the value of ML models in preoperative predictions of sperm retrieval. These models support better patient counseling and surgical decision-making. They also offer more precise identification of patients most likely to benefit from m-TESE (Jamalirad et al., 2025). For successful fertilization and proper embryo development, it is most important to assess the maturity of oocytes. Mature oocytes in metaphase II have a higher fertilization rate compared to immature oocytes (Sciorio et al., 2024). For oocyte donation cycles during oocyte freezing for fertility preservation, morphology evaluation is very useful. It can serve as a tool to explain very poor treatment results (Nikiforov et al., 2022). Morphokinetics, which relies on time-lapse imaging, allows for continuous observation of embryonic development (Anagnostopoulou et al., 2022). The predictive ability of time-lapse monitoring (TLM) selection algorithms is affected by patient characteristics, the quality of the data included in the analysis and the statistical methods used (van Marion et al., 2023). Infertility clinics are using TLM as an attempt to improve their ability to select embryos with the highest potential for implantation used. Many markers of embryo morphokinetics have been incorporated into decision-making algorithms for embryo (de)selection (Bayram et al., 2024). Pre-implantation genetic testing for aneuploidy (PGT-A) using whole-genome amplification (WGA) combined with next-generation sequencing (NGS) techniques has made it possible to create opportunities to identify embryo biopsies that are dictated by mosaicism (Surrey, 2021).

## 6 Overview of clinical case studies

Extensive laboratory studies and clinical cases widely present in scientific literature highlight the immense significance of embryonic engineering and fertility research in modern medicine (Francés-Herrero et al., 2022).

In 2015, a Chinese research team under the supervision of Junjiu Huang edited the HBB gene, which encodes beta-globin, in non-viable tripronuclear embryos using the CRISPR-Cas9 genetic engineering method (Liang et al., 2015). This breakthrough paved the way for further research, bringing therapeutic prospects of this method closer to reality. Just a year later, on 28 October 2016, Lu You at Sichuan University in Chengdu, as part of clinical trials, administered modified cells to a patient suffering from aggressive non-small-cell lung cancer. These cells were previously extracted from the patient’s blood, and the defective genes encoding the PD-1 immunoglobulin were removed using CRISPR-Cas9 technology (Cyranoski, 2016a; 2016b). This study marked a milestone in genetic engineering, offering hope for cancer patients who do not respond to conventional oncological treatments. In 2018, a team of scientists from the University of California, Berkeley, presented a study in which they successfully cured a mouse with fragile X syndrome using CRISPR-Cas9 by modifying genetic material present in its brain (Lee et al., 2018). They employed gold nanoparticle ions as carriers for the DNA-cutting enzyme, known as CRISPR-Gold, which led to changes in the expression of the metabotropic glutamate receptor 5 (mGluR5) gene. This resulted in behavioral improvements related to autism spectrum disorder, associated with the aforementioned condition. These studies revolutionized modern medicine, opening up new possibilities for treating neurological diseases through safe gene editing in brain tissue. In November of the same year, the first twins with genes modified using the CRISPR-Cas9 method were born. This technology was utilized to modify the CCR5 gene in embryos obtained via *in vitro* fertilization, aiming to make them resistant to HIV infection. This experiment sparked significant controversy while simultaneously opening further possibilities for therapeutic interventions in the human genome (Raposo, 2019). Additionally, CRISPR-Cas9 has increased the effectiveness of combating hereditary heart diseases by repairing the MYBPC3 gene responsible for hypertrophic cardiomyopathy (Nie et al., 2023). CRISPR-Cas9 has also revolutionized the field of transplantation. Tissues grown from pigs for xenotransplantation purposes, in which the embryonic genome was previously modified using this method, demonstrate a lower risk of rejection by the human host. This technique significantly surpasses traditionally obtained transplant tissues through *in vitro* methods or ICSI (Briski et al., 2024).

In recent years, numerous studies have focused on the future of fertility in women suffering from cancer or hormonal disorders such as PCOS, which prevent conception and lead to permanent infertility. To address this, oocytes were collected from patients with PCOS and allowed to grow further using *in vitro* methods. Subsequently, cryotechnology was employed to freeze them, enabling their future use for fertilization and pregnancy through embryo transfer (El Hachem et al., 2014; Vuong et al., 2020). This technique resulted in successful births for previously infertile women with PCOS who underwent the procedure (Li et al., 2016). This method has also gained popularity among individuals using it for non-medical reasons. Published findings indicate that oocyte cryopreservation via vitrification does not impair their development or quality after thawing. It’s equally satisfactory effectiveness has opened a new pathway for reproductive possibilities and further research in this field (Garcia-Velasco et al., 2013). Similarly, a successful live birth was achieved for a woman with stage IV aggressive Hodgkin’s lymphoma undergoing gonadotoxic chemotherapy. Before treatment, not only single ovarian cells but an entire fragment of ovarian tissue was extracted and cryopreserved for safe storage. Several years later, after completing cancer treatment and discontinuing hormone replacement therapy, the patient was diagnosed with ovarian failure. An autotransplant of the previously preserved ovarian tissue was performed to restore its normal functions and enable pregnancy. Over the following months, the ovary gradually regained its functions, allowing the patient to conceive naturally (Donnez et al., 2004). This case opened numerous new perspectives for preventing the adverse effects of infertility in women undergoing aggressive oncological treatments, giving them renewed hope for having children. Over the past few years, scientists have examined various methods of collecting ovarian tissue, embryos, and oocytes subjected to freezing processes in greater detail. These studies have shown promising results while emphasizing the continued necessity of research and exploration of specific techniques to assess their real potential, benefits, as well as possible complications and failures (Ní Dhonnabháin et al., 2022). Each of these methods should be individually tailored to the patient, considering both its advantages and limitations.

Male fertility restoration techniques utilizing cryotherapy have also gained popularity recently. Available processes include sperm freezing for future IVF, allowing patients to become biological fathers (Tran et al., 2022). However, this procedure is not feasible for young boys undergoing gonadotoxic therapy due to immature sperm before puberty. As a result, efforts have been made to freeze testicular tissue containing spermatogonia, representing a promising solution that has initiated further progress and development in this field (Practice Committee of the American Society for Reproductive Medicine, 2019; Jensen et al., 2022).

During fertility research, a clinical research team from Chongqing, China, emerged with significant findings. Between 2019 and 2022, they gathered a group of men suffering from primary infertility caused by morphological abnormalities in sperm flagella. These patients were diagnosed with defective variants of the DNAH1 gene, confirming its role in sperm flagellar structure. They were offered the ICSI method to achieve fertilization and subsequent pregnancy. This approach led to several successful births, demonstrating the potential of this technique in treating male infertility caused by sperm flagellar abnormalities (Long et al., 2024). This study represents yet another milestone, encouraging further exploration and advancements in human reproductive science. The ICSI procedure mentioned above can also be combined with cryotechnology methods, utilizing thawed embryos that were previously frozen after their initial retrieval. The combination of these two procedures has resulted in a higher live birth rate and a lower miscarriage rate, as demonstrated in a clinical retrospective study conducted by the First Affiliated Hospital of Anhui Medical University (Wang et al., 2018).

Peroxisome proliferator-activated receptors (PPARs) have also proven their significance. They influence various processes occurring in the body, including metabolic, inflammatory, and cellular differentiation mechanisms, which play a crucial role in reproductive processes. Their interaction ensures homeostasis in the human body and regulates gene expression (Berger and Moller, 2002). One of the fertility treatment strategies for the previously mentioned PCOS is based on these receptors. They participate in the synthesis of steroid hormones in the ovary, directly inducing aromatase through their role in steroidogenesis. As a result, modulating their activity may yield the desired clinical effect (Suriyakalaa et al., 2021). The importance of PPAR receptors in determining fertility is further emphasized by research on the toxicity of Mn3O4 in the male reproductive system through their signaling pathways (Zhang et al., 2020). These receptors also regulate spermatogenesis, directly affecting sperm function and quality. Systemic metabolic disorders can lead to the dysregulation of the PPARγ signaling pathway, which exists as a heterodimer with the retinoid X receptor (RXR) and is activated by various natural and synthetic ligands such as prostaglandin derivatives, PUFAs, and thiazolidinediones, all of which significantly impair male fertility. Biological and pharmacological interventions, such as the use of PPARγ agonists, constitute an important therapeutic strategy for men struggling with metabolic-related fertility issues (Santoro et al., 2020).

A review of clinical cases highlights interdisciplinary collaboration among scientists as a necessity for treating male infertility (Dissanayake et al., 2019). Personalized treatment strategies and the integration of various therapeutic methods contribute to increasingly successful clinical outcomes. Advances in andrology also impact the quality and course of pregnancy (Calogero et al., 2023).

Also noteworthy are new methods and ideas, not yet fully implemented in common clinical and laboratory practice, which create a number of new opportunities and avenues for development in germline engineering in the course of fertility research.

Female infertility may soon become a thing of the past thanks to innovative 3D printing methods (Alzamil et al., 2021). Regenerative therapies are being developed for women with impaired ovarian function, for example, following aggressive chemotherapy. Research is focused on obtaining various materials that can enable the integration of ovarian cells to ensure stable growth and restore full tissue function, achieving proper folliculogenesis and endocrine efficiency with the help of printed scaffolds (Laronda, 2020; Nair et al., 2024). However, interdisciplinary collaboration between biotechnologists, genetic engineers, biologists, and clinical physicians is necessary to develop fully functional and effective therapeutic models that are safe for clinical practice (Ferronato et al., 2024). Virtual printing also demonstrates potential in treating male infertility. With its assistance, organoids related to male testes have been successfully created (Patrício et al., 2023). These organoids can serve as *in vivo* models formed using printed scaffolds and specialized culture conditions. The recreated natural testicular niche provides an excellent foundation for studying the process of spermatogenesis through the long-term maintenance of early-stage germ cells and controlling their entry into meiosis. Moreover, this structure represents another starting point for the further development of regenerative male fertility therapies (Ghanbari et al., 2020; Richer et al., 2021).

Preimplantation diagnostics is also a crucial aspect that cannot be overlooked in fertility research. Its precise execution significantly increases the effectiveness of reproductive therapies. Genetics also plays a key role in this process (Yahaya et al., 2020). This has been demonstrated by scientists from Shandong, who conducted a cohort retrospective study between 2014 and 2022 at the local Center for Reproductive Medicine, proving the efficacy of preimplantation genetic testing focused on monogenic disorders (PGT-M) related to hereditary nephropathy in preventing the inheritance of this disease. This procedure has proven effective in successfully delivering children free of monogenic kidney pathology in affected couples. This study highlights the potential of both genetics and preimplantation diagnostics, offering the possibility of eliminating hereditary diseases and giving affected individuals a chance for healthy offspring (Liu et al., 2025). A groundbreaking advancement in this field has also been the development of diagnostic systems based on AI. Using only a single time-lapse image, an AI algorithm can select embryos with the best potential for further development. In an automated manner, with remarkable efficiency, it surpasses the work of contemporary embryologists (Kanakasabapathy et al., 2020). However, this study focuses solely on blastocyst development, though it serves as a breakthrough foundation for further research, analyzing pregnancy efficiency and live birth rates. AI sequences based on time-lapse imaging have also been developed, allowing for a thorough analysis to select embryos with the highest implantation potential based on their morphological characteristics and developmental dynamics (Berntsen et al., 2022). These approaches have shown promising results, which are crucial for future clinical applications. However, the use of AI in medicine raises numerous ethical concerns, making it essential to develop models that explain AI-driven decision-making. Scientists emphasize the importance of AI transparency to ensure collaboration with clinical physicians and facilitate its broader application in key decision-making processes (Urcelay et al., 2023).

Additionally, emerging studies analyze the future potential of embryonic engineering methods and fertility research concerning reproduction in space. Researchers highlight the importance of exploring these technologies, which may ensure fertility for astronauts, particularly during long-term missions, and serve as a cornerstone for development in the era of space exploration. The key challenges include the unique extraterrestrial conditions and the safety of conducted procedures (Chaplia et al., 2024; Sharma et al., 2024).

## 7 Ethical and legal aspects

Interdisciplinary collaboration, the continuous advancement of science and technology, and the growing interest and awareness of society contribute to the increasing progress in fields related to fertility and genetic engineering of embryos. However, these research directions also give rise to numerous controversies and contradictions, often challenging well-established ethical and legal norms.

A survey conducted in Japan in 2019 found that, on average, one in four respondents strongly opposed genome editing for research purposes, regardless of its application. In contrast, approximately half of the respondents expressed acceptance of germline genome intervention for research on diseases, such as the elimination of chronic illnesses. A slightly smaller proportion of respondents approved genome editing in basic research aimed at gaining biological knowledge. The scientific community and experts participating in political and bioethical debates demonstrated a higher percentage of approval for genome intervention. However, their assumptions and views are not always fully understood by the general public (Akatsuka et al., 2023). A global survey on human genome editing was also conducted recently through social media. This approach allowed researchers to reach a highly diverse, multicultural group and gather their opinions. Nearly 60% of respondents supported gene editing for the purpose of eliminating life-threatening and debilitating diseases. However, this approval dropped to approximately 40% when the genetic modifications were intended for non-medical purposes. The study also highlighted the influence of ethical and moral perspectives, particularly in relation to upbringing and religious beliefs. For example, individuals identifying as Christians were significantly more likely than others to oppose any form of gene editing. On the other hand, Muslim respondents showed greater support for genetic modifications for non-medical purposes compared to non-religious respondents (McCaughey et al., 2016). An article published on 26 July 2018, by the Pew Research Center presented the views of Americans on gene editing in children. A statistical majority of U.S. citizens expressed approval of genome editing for the elimination of hereditary congenital diseases. However, this percentage declined when the modifications aimed to reduce the risk of severe illnesses over a lifetime, although approval still remained dominant. Notably, an overwhelming 80% of respondents expressed strong disapproval of gene editing for the purpose of creating “enhanced” children, such as those with higher intelligence quotients. At this stage, survey participants accused such technological advancements of being a severe misuse of medical science. This sentiment was also overwhelmingly reflected in responses regarding opinions on further research involving human embryos, with two out of three Americans rejecting such practices. Additionally, religious Americans and those without scientific knowledge of the subject expressed greater reluctance toward genetic modification methods. Furthermore, the study revealed that most Americans primarily perceive negative aspects of gene editing, overlooking or failing to recognize its potential benefits. This tendency is more prevalent among individuals with lower awareness and knowledge in this field (Funk and Hefferon, 2018).

The wide range of public opinions, coupled with significant concerns about the integrity of the human genome and the diverse consequences of its alteration, necessitates the development of a standardized, generalized, and transparent set of ethical and legal regulations, placing considerable responsibility on governing institutions (Delhove et al., 2020).

Over recent decades, certain legal guidelines have already been established concerning technological advancements in medicine, based on ethical principles (Andrews, 1986; Wilson, 2018). However, with recent developments, genetic engineering has advanced far beyond its previous scope, securing a position in increasingly widespread clinical applications, as demonstrated by the extensive use of CRISPR-Cas9 methods (Vassena et al., 2016; Khan et al., 2018). This progress carries numerous implications and moral dilemmas (Bilir et al., 2020). Genetic alterations may be perceived as a limitation of the embryo’s autonomy, and consequently, that of the individual it will develop into. A real risk exists regarding the transmission of uncontrolled hereditary mutations to future offspring or the emergence of unintended mutations in other parts of the genome, known as off-target effects (Mulvihill et al., 2017). The inability to fully influence subsequent embryonic genetic processes may also result in mosaicism (Mehravar et al., 2019; Mohiuddin et al., 2022). Experts emphasize the importance of transparency and interdisciplinary collaboration to ensure maximum safety and public acceptance of embryonic engineering methods, supported by full awareness of both the benefits and potential complications. More cohesive, preferably internationally established, ethical and legal guidelines are necessary to continue research and improve existing techniques. This would help eliminate any ethical uncertainties regarding genetic interventions while ensuring the highest possible level of protection for embryonic eugenics (European Commissionand European Group on Ethics in Science and New Technologies, 2018).

Ethics is an integral part of our lives, as well as the field of medicine in the broad sense, shaping and responding to moral dilemmas. It is important that it be constantly subjected to criticism and review, continuously evaluated and accepted not only by the professional community in general, but also by society, in order to prevent its bias (Hofmann, 2025). This complexity can lead to an overwhelming number of dilemmas, particularly concerning advancements in research and the emergence of new scientific technologies in medicine. However, the absence of progress is not a desirable predictive factor for humanity. Morality dictates that we should utilize emerging solutions to foster future improvements, yet it is crucial to consider safety concerns and existing risks. This necessity calls for continuous discussions, meetings, and the constant refinement of established guidelines to ensure the most rational approach—one that preserves as many ethical values as possible while also being grounded in scientific convictions based on obtained results (Harris, 2010). It is important to emphasize that law does not always align with ethics. While legal frameworks often derive from ethical principles, in some cases, they can be entirely separate, failing to conform to the moral expectations of the society they govern (Fuchs, 2024).

Existing legal norms vary significantly between countries, leading to considerable controversy and inconsistencies on the international stage. Researchers from the United States highlight the difficulties associated with embryonic research, pointing out the wide divergence in legal standards across different states. In some regions, fundamental research activities are prohibited, with restrictions such as the time frame within which embryo intervention is permissible—an issue that poses no legal challenges in other parts of the country. Furthermore, federal law does not distinguish significantly between an embryo and a fetus, despite this distinction being highly relevant in scientific contexts. Additional regulations, such as the Dickey-Wicker Amendment, attempt to address these issues, yet many inconsistencies remain. These regulations fail to generalize guidelines concerning embryonic research, necessitating the case-by-case evaluation of each experiment’s principles and funding. Consequently, financial support for such projects often comes from private entities, as they are then subject only to state-specific legal restrictions. Researchers continue to call for updates and standardization of current legal norms to facilitate the smoother development of embryonic engineering and the acquisition of more reliable knowledge about embryos (Matthews and Morali, 2022). The legal-scientific conflict is also evident in Europe, where the permissibility of human embryo research varies by country, with some nations allowing it while others impose strict prohibitions. Germany has one of the most restrictive legal frameworks concerning reproductive medicine, with numerous limitations and prohibitions on embryo exploration (Burfoot and Waldschmidt, 2019). Only basic procedures enabling pregnancy through *in vitro* methods are permitted (Advena-Regnery et al., 2018). European policies are subject to ongoing changes and numerous debates, yet they do not always keep pace with the rapid advancements in reproductive medicine. France serves as an example of this discrepancy; although embryonic cell research has been legally permitted since 2013, it remains heavily constrained by extensive legal restrictions. The primary ethical concern revolves around the destruction of embryos and the lack of control over their further development. Over the years, various methods for acquiring embryonic cells for research have been considered, including obtaining them from couples undergoing IVF who consent to donating non-viable embryos designated for elimination. A persistent debate has emerged over whether these cells are merely biological constructs or constitute a human being (Duguet et al., 2018). In contrast, China does not legally recognize human embryos as human beings, resulting in fewer legislative restrictions or prohibitions concerning them (Raposo and Ma, 2020). However, in 2014, during a court case concerning frozen embryos, it was acknowledged that embryos represent a special structure with the potential to develop into a human life, necessitating a respectful and moral approach to their handling. The destruction of embryos is therefore strongly condemned and regarded as unethical. Nonetheless, the lack of legal recognition of embryos as human beings has facilitated significant advancements and widespread research on embryonic cell lines. However, in 2019, the Chinese scientists responsible for the birth of the world’s first genetically edited children the previous year were sentenced to heavy fines and 3 years in prison. Despite the country’s leniency toward embryonic research, China does not permit genetic interventions in living human organisms. Due to legal loopholes, the verdict was issued solely for unauthorized medical practices, but it served as a catalyst for stricter legal reforms. The Civil Code, established in 2020, mandated compliance with all existing legal standards while explicitly prohibiting interference with fundamental bioethical and moral principles. The law also strictly banned reproductive human cloning (Peng et al., 2020). Despite differences in legal regulations, Chinese researchers and those responsible for embryonic studies worldwide are guided by the same moral and ethical norms, as well as concerns about issues such as eugenics in developing organisms. These ethical considerations resonate across societies worldwide (Zhai et al., 2016). Thus spoken, it is imperative to establish universally accepted international regulations and guidelines to ensure the safe and socially acceptable advancement of human reproductive medicine.

## 8 Anticipated challenges and future directions–a discussion

We are undertaking a literature review to determine the current state of knowledge on infertility and potential methods of addressing this issue. The observed correlation of multiple factors contributing to difficulties in natural conception highlights the need to explore new therapeutic possibilities from an interdisciplinary perspective. Our aim is to assess the impact of developments in various fields on discoveries in this area and their potential societal implications. Reports from professionals and individuals directly affected by infertility from 40 countries have raised the question of whether it is even possible to define strategic goals in infertility research. Respondents emphasized the importance of focusing, among other things, on environmental factors and comorbidities (Duffy et al., 2021). Indeed, of the more than 186 million people affected by infertility, the majority are from developing regions (Vander Borght and Wyns, 2018). The findings support the significant impact of environmental factors on reproductive health. These include elevated temperatures beyond optimal ranges, atmospheric pollution such as tobacco smoke, and heavy metals with high atomic mass. Notably, many of these exposures can be limited by modifying the behaviors of at-risk individuals (Kumar and Singh, 2022; Wdowiak et al., 2024). As a result, addressing the infertility issue in an interdisciplinary manner also encompasses domains unrelated directly to medicine, as the mitigation of the aforementioned factors should be overseen by specialists in environmental protection and remediation. Researchers highlight the need to develop efficient, modern methods—such as the use of microorganisms in bioremediation and detoxification processes (Nnaji et al., 2023). Alongside biological determinants, economic factors are also recognized. Changes in this area began in the early 20th century and have led to a dramatic decline in reproductive rates in some industrialized regions, falling below replacement levels (Skakkebæk et al., 2021). The perception of a higher reproductive age in women due to changing lifestyles and social norms, combined with the aging of many societies, increases the likelihood of conception difficulties (Bala et al., 2020). Geographic location, considered broadly as part of environmental factors, further translates into documented inequalities in access to infertility therapies due to the economic status of individual countries. This remains the case despite the fact that male infertility diagnostics are relatively simple and inexpensive. This leads to the conclusion that certain restrictions limit access to assistance in cases of infertility and generate stress among potential patients. Passet-Wittig and Greil have urged medical professionals to acknowledge this and undertake efforts to expand support in this field (Passet-Wittig and Greil, 2021; Di Bello et al., 2022). Researchers emphasize the importance of interpreting physiological changes through a psychological lens in future studies and the need to develop standardized tools to assess the mental and emotional state of patients, which could serve as valuable support in infertility treatment (Zhu et al., 2022). The involvement of nutrition specialists is also crucial. A balanced, unprocessed diet with a low glycemic index, sometimes combined with supplementation of vitamins (e.g., B12) and minerals (e.g., iodine, iron), not only enhances the effectiveness of ART but also directly supports fertility (Łakoma et al., 2023). Geneticists also advocate for interdisciplinary cooperation. Considering the impact of environmental factors on sperm epigenome—such as DNA methylation, which affects fertilization—they argue for closer integration of epigenetic analysis into clinical practice. Enhanced tools for phenotype analysis and genomic testing may significantly expand future knowledge in this field (Salas-Huetos and Aston, 2021). Genetic causes of male infertility are confirmed in approximately 4% of cases (Houston et al., 2021). Infertility can also result directly from various diseases or as a side effect of treatment. Relevant questions are being raised about which conditions and therapies contribute to infertility. Massarotti et al. examined this issue in the context of multiple sclerosis. Due to the inability to eliminate the disease’s effect on fertility and its association with sexual dysfunctions, they underscore the necessity of collaboration among specialists from multiple fields. They point to a shortage of data and the need to document the impact of novel immunotherapies on semen quality. They propose selecting treatments that minimize negative reproductive consequences (Massarotti et al., 2021). Infectious diseases also play a significant role. In the case of HIV, the infection itself reduces sperm motility and ejaculate volume, and antiretroviral therapy further compromises semen parameters. Emerging techniques, such as sperm washing, show promise and may become effective alternatives in the future, though they still require refinement (Guo et al., 2024). Research directions in the field of infertility appear promising. Currently utilized techniques are sufficiently advanced to serve as a foundation for innovative approaches. ART has contributed to a new understanding of embryological processes by elucidating the correlation between the oocyte and the sperm. The future may bring promising developments for azoospermic men through the use of stem cells and other assisted reproduction methods (Schlegel et al., 2021). *In vitro* culture of iPSC, reprogrammed from somatic cells, has the potential to generate reproductive cells. Scientific advancements have reached a point where genetic material can even be modified to eliminate identified anomalies (Gul et al., 2024). Undoubtedly, these advancements will be supported by digital systems and modern technologies. AI already supports medical practice and is expected to play an integral role in future developments. Beyond semen analysis, diagnosis, and therapeutic recommendations, AI is projected to contribute to ultrasound analysis of semen and mTESE procedures (Venishetty et al., 2024). However, the effective use of AI depends on access to vast amounts of data, which must be efficiently processed and analyzed. Medenica et al. emphasize the importance of improving data accessibility, viewing this as a prerequisite for integrating AI into routine clinical practice (Medenica et al., 2022). To validate the efficacy of modern techniques, numerous randomized trials and registries are needed. These may confirm the greater effectiveness of integrating human expertise with AI systems compared to either approach alone (Abdullah et al., 2023). Future research is expected to focus heavily on the use of ultrasound to identify sperm production sites, as well as the selection and analysis of sperm through CASA (Diaz et al., 2022).

**FIGURE 3:**
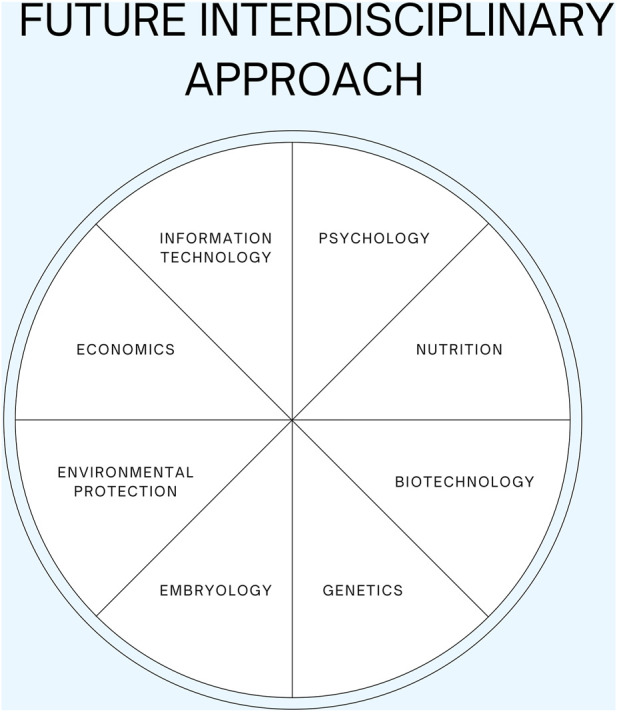
Future interdisciplinary approach.

Our reflections suggest that an effective fight against infertility requires a well-prepared multidisciplinary *task force* capable of conducting comprehensive diagnostics and implementing advanced therapies. Ideally, this team would include experts not only in medicine but also in environmental protection and socio-economic development (Yopo Díaz and Watkins, 2025). We believe that the commitment and collaboration of professionals from these domains could lead to transformative progress, benefiting both patients and healthcare providers. Improvements in this field may also contribute to better mental health outcomes, reinforcing the need for a holistic and interdisciplinary approach to infertility and the exploration of new treatment pathways. Figure 3 illustrates the potential directions for a future interdisciplinary approach.

## 9 Conclusion

The key to developing new, potentially more widely accessible therapeutic interventions is the involvement of interdisciplinary teams of specialists, who, in addition to medical determinants, also take into account economic, environmental, and psychosocial factors, due to the impact of environmental pollution and unhealthy lifestyles on fertility. Ethical and legal controversies require coherent, international regulations.

Future perspectives indicate progress in the fields of biotechnology and gene therapies, supported by AI; however, the collection of relevant data is essential for this purpose, with registries and multicenter studies serving as potential sources.

## References

[B1] Abdullah K. A. L. Atazhanova T. Chavez-Badiola A. Shivhare S. B. (2023). Automation in ART: paving the way for the future of infertility treatment. Reprod. Sci. 30, 1006–1016. 10.1007/S43032-022-00941-y 35922741 PMC10160149

[B2] Advena‐Regnery B. Dederer H. Enghofer F. Cantz T. Heinemann T. (2018). Framing the ethical and legal issues of human artificial gametes in research, therapy, and assisted reproduction: a German perspective. Bioethics 32, 314–326. 10.1111/bioe.12433 29878465 PMC6001525

[B3] Agarwal A. Baskaran S. Parekh N. Cho C. L. Henkel R. Vij S. (2021). Male infertility. Lancet 397, 319–333. 10.1016/S0140-6736(20)32667-2 33308486

[B4] Aitken R. J. (1995). Free radicals, lipid peroxidation and sperm function. Reprod. Fertil. Dev. 7 (4), 659–668. 10.1071/RD9950659 8711202

[B5] Akatsuka K. Hatta T. Sawai T. Fujita M. (2023). Genome editing of human embryos for research purposes: japanese lay and expert attitudes. Front. Genet. 14, 1205067. 10.3389/fgene.2023.1205067 37424733 PMC10324961

[B6] Aktuna S. Unsal E. Ozer L. Aydin M. Baltaci V. (2019). 73. Simultaneous pgt-m applications for multiple genetic conditions. Reprod. Biomed. Online 39, e72. 10.1016/j.rbmo.2019.04.126

[B7] Alzamil L. Nikolakopoulou K. Turco M. Y. (2021). Organoid systems to study the human female reproductive tract and pregnancy. Cell Death Differ. 28, 35–51. 10.1038/s41418-020-0565-5 32494027 PMC7852529

[B8] Anagnostopoulou C. Maldonado Rosas I. Singh N. Gugnani N. Chockalingham A. Singh K. (2022). Oocyte quality and embryo selection strategies: a review for the embryologists, by the embryologists. Panminerva Med. 64, 171–184. 10.23736/S0031-0808.22.04680-8 35179016

[B9] Andrews L. B. (1986). The legal status of the embryo. Loy L Rev. 32, 357–409. 11658916

[B10] Anwar S. Anwar A. (2016). Scient open access exploring the world of science infertility: a review on causes, treatment and management. Womens Health Gynecol. 5, 2–5.

[B11] Aoyama N. Kato K. (2020). Trophectoderm biopsy for preimplantation genetic test and technical tips: a review. Reprod. Med. Biol. 19, 222–231. 10.1002/RMB2.12318 32684821 PMC7360970

[B12] Aydin M. Aktuna S. Unsal E. Ozer L. Baltaci V. (2019). 43. NGS BASED PGT-A/PGT-SR: DATA FROM >7000 EMBRYOS. Reprod. Biomed. Online 39, e53. 10.1016/j.rbmo.2019.04.096

[B13] Bachelot G. Dhombres F. Sermondade N. Hamid R. H. Berthaut I. Frydman V. (2023). A machine learning approach for the prediction of testicular sperm extraction in nonobstructive azoospermia: algorithm development and validation study. J. Med. Internet Res. 25, e44047. 10.2196/44047 37342078 PMC10337455

[B14] Bala R. Singh V. Rajender S. Singh K. (2020). Environment, lifestyle, and female infertility. Reprod. Sci. 28, 617–638. 10.1007/S43032-020-00279-3 32748224

[B15] Balaban B. Urman B. Alatas C. Mercan R. Mumcu A. Isiklar A. (2002). A comparison of four different techniques of assisted hatching. Hum. Reprod. 17, 1239–1243. 10.1093/HUMREP/17.5.1239 11980745

[B16] Bauché F. Fouchard M. H. Jégou B. (1994). Antioxidant system in rat testicular cells. FEBS Lett. 349, 392–396. 10.1016/0014-5793(94)00709-8 8050602

[B17] Baynosa J. Westphal L. M. Madrigrano A. Wapnir I. (2009). Timing of breast cancer treatments with oocyte retrieval and embryo cryopreservation. J. Am. Coll. Surg. 209, 603–607. 10.1016/J.JAMCOLLSURG.2009.08.006 19854400

[B18] Bayram A. Elkhatib I. Kalafat E. Abdala A. Ferracuti V. Melado L. (2024). Steady morphokinetic progression is an independent predictor of live birth: a descriptive reference for euploid embryos. Hum. Reprod. Open 2024, hoae059. 10.1093/HROPEN/HOAE059 39507416 PMC11540439

[B19] Beghini D. G. Kasai-Brunswick T. H. Henriques-Pons A. (2024). Induced pluripotent stem cells in drug discovery and neurodegenerative disease modelling. Int. J. Mol. Sci. 25, 2392. 10.3390/IJMS25042392 38397069 PMC10889263

[B20] Benhabbour S. R. (2025). 3D-printed intravaginal ring for infertility treatment. Nat. Rev. Bioeng. 3, 104–105. 10.1038/s44222-025-00272-y

[B21] Berger J. Moller D. E. (2002). The mechanisms of action of PPARs. Annu. Rev. Med. 53, 409–435. 10.1146/annurev.med.53.082901.104018 11818483

[B22] Berntsen S. Laivuori H. La Cour Freiesleben N. Loft A. Söderström-Anttila V. Oldereid N. B. (2021). A systematic review and meta-analysis on the association between ICSI and chromosome abnormalities. Hum. Reprod. Update 27, 801–847. 10.1093/HUMUPD/DMAB005 33956940

[B23] Berntsen J. Rimestad J. Lassen J. T. Tran D. Kragh M. F. (2022). Robust and generalizable embryo selection based on artificial intelligence and time-lapse image sequences. PLoS One 17, e0262661. 10.1371/journal.pone.0262661 35108306 PMC8809568

[B24] Biggers J. D. (1991). Walter heape, FRS: a pioneer in reproductive biology. Centenary of his embryo transfer experiments. Reproduction 93, 173–186. 10.1530/JRF.0.0930173 1920287

[B25] Bilir E. Vatanoğlu Lutz E. E. Özgönül M. L. (2020). Ethical and scientific issues of gene-edited twin by clustered regularly interspaced short palindromic repeats Cas9 technology. J. Turkish-German Gynecol. Assoc. 21, 138–139. 10.4274/jtgga.galenos.2019.2019.0153 31927815 PMC7294836

[B26] Briski O. La Motta G. E. Ratner L. D. Allegroni F. A. Pillado S. Álvarez G. (2024). Comparison of ICSI, IVF, and *in vivo* derived embryos to produce CRISPR-Cas9 gene-edited pigs for xenotransplantation. Theriogenology 220, 43–55. 10.1016/j.theriogenology.2024.02.028 38471390

[B27] Bulletti C. Franasiak J. M. Busnelli A. Sciorio R. Berrettini M. Aghajanova L. (2024). Artificial intelligence, clinical decision support algorithms, mathematical models, calculators applications in infertility: systematic review and Hands-On digital applications. Mayo Clin. Proc. Digit. Health 2, 518–532. 10.1016/J.MCPDIG.2024.08.007 40206524 PMC11975849

[B28] Burfoot A. Waldschmidt A. (2019). in Encyclopedia of reproductive technologies - legislation—germany. Editor Burfoot A. (New York: Routledge). 10.4324/9780429037399

[B29] Calogero A. E. Cannarella R. Agarwal A. Hamoda T. A.-A. A.-M. Rambhatla A. Saleh R. (2023). The Renaissance of Male infertility management in the golden age of andrology. World J. Mens. Health 41, 237–254. 10.5534/wjmh.220213 36649928 PMC10042649

[B245] Cavoretto P. Candiani M. Giorgione V. Inversetti A. Abu-Saba M. M. Tiberio F. (2018). Risk of spontaneous preterm birth in singleton pregnancies conceived after IVF/ICSI treatment: meta-analysis of cohort studies. Ultrasound Obstet Gynecol 51, 43–53. 10.1002/uog.18930 29114987

[B246] Cavoretto P. I. Giorgione V. Sotiriadis A. Vigano P. Papaleo E. Galdini A. (2020). IVF/ICSI treatment and the risk of iatrogenic preterm birth in singleton pregnancies: systematic review and meta-analysis of cohort studies. J. Matern. Fetal Neonatal. Med. 35, 1987–1996. 10.1080/14767058.2020.1771690 32498576

[B30] Campbell-Forde M. Drew E. Odia R. Cawood S. Douglas K. Seshadri V. (2024). P-557 The contrtbution of PGT-A to PGT-M and PGT-SR clinical outcomes – A single centre retrospective analysis. Hum. Reprod. 39, deae108.894. 10.1093/HUMREP/DEAE108.894

[B247] Chaplia O. Mathyk B. A. Nichols-Burns S. Basar M. Halicigil C. (2024). Beyond Earth’s bounds: navigating the frontiers of Assisted Reproductive Technologies (ART) in space. Reprod. Biol. Endocrinol. 22, 123. 10.1186/s12958-024-01290-y 39394617 PMC11468284

[B31] Chen L. Yang T. Zheng Z. Yu H. Wang H. Qin J. (2018). Birth prevalence of congenital malformations in singleton pregnancies resulting from *in vitro* fertilization/intracytoplasmic sperm injection worldwide: a systematic review and meta-analysis. Arch. Gynecol. Obstet. 297, 1115–1130. 10.1007/S00404-018-4712-X 29497821

[B32] Chen T. Fan D. Wang X. Mao C. Chu Y. Zhang H. (2022). ICSI outcomes for infertile men with severe or complete asthenozoospermia. Basic Clin. Androl. 32, 6–8. 10.1186/S12610-022-00155-x 35382740 PMC8981622

[B33] Christianti F. Y. Legiran L. (2024). *In vitro* fertilization as an option for couples with genetic disorders. Clin. Exp. Reprod. Med. 52, 1–7. 10.5653/CERM.2023.06667 38853127 PMC11900667

[B34] Chu K. Y. Nassau D. E. Arora H. Lokeshwar S. D. Madhusoodanan V. Ramasamy R. (2019). Artificial intelligence in reproductive urology. Curr. Urol. Rep. 20, 52. 10.1007/s11934-019-0914-4 31353422

[B35] Cimadomo D. Capalbo A. Ubaldi F. M. Scarica C. Palagiano A. Canipari R. (2016). The impact of biopsy on human embryo developmental potential during preimplantation genetic diagnosis. Biomed. Res. Int. 2016, 7193075. 10.1155/2016/7193075 26942198 PMC4749789

[B36] Coll L. Parriego M. Boada M. Devesa M. Arroyo G. Rodríguez I. (2018). Transition from blastomere to trophectoderm biopsy: comparing two preimplantation genetic testing for aneuploidies strategies. Zygote 26, 191–198. 10.1017/S0967199418000084 29798732

[B37] Cox G. F. Bürger J. Lip V. Mau U. A. Sperling K. Wu B. L. (2002). Intracytoplasmic sperm injection may increase the risk of imprinting defects. Am. J. Hum. Genet. 71 (1), 162–164. 10.1086/341096 12016591 PMC384973

[B38] Cozzolino M. Cecchino G. N. Garcia Velasco J. A. Pellicer N. Galliano D. Pellicer A. (2023). Preimplantation genetic testing for aneuploidy is not related to adverse obstetric and neonatal outcomes in singleton pregnancies. Hum. Reprod. 38, 1621–1627. 10.1093/HUMREP/DEAD123 37336546

[B39] Cutting E. Horta F. Dang V. van Rumste M. M. E. Mol B. W. J. (2023). Intracytoplasmic sperm injection *versus* conventional *in vitro* fertilisation in couples with males presenting with normal total sperm count and motility. Cochrane Database Syst. Rev. 2023. 10.1002/14651858.CD001301.PUB2 37581383 PMC10426261

[B40] Cybulska N. (2019). Uniwersytet medyczny im. piastów śląskich we wrocławiu. Available online at: https://ppm.edu.pl.

[B41] Cyranoski D. (2016a). Chinese scientists to pioneer first human CRISPR trial. Nature 535, 476–477. 10.1038/nature.2016.20302 27466105

[B42] Cyranoski D. (2016b). CRISPR gene-editing tested in a person for the first time. Nature 539, 479. 10.1038/nature.2016.20988 27882996

[B43] de Miguel-Beriain I. (2015). The ethics of stem cells revisited. Adv. Drug Deliv. Rev. 82 (83), 176–180. 10.1016/J.ADDR.2014.11.011 25446134

[B44] de Rycke M. Berckmoes V. de Vos A. de Voorde S. V. Verdyck P. Verpoest W. (2020). Preimplantation genetic testing: clinical experience of preimplantation genetic testing. Reproduction 160, A45–A58. 10.1530/REP-20-0082 33112789

[B45] de Santiago I. Polanski L. (2022). Data-driven medicine in the diagnosis and treatment of infertility. J. Clin. Med. 11, 6426. 10.3390/JCM11216426 36362653 PMC9654112

[B46] De Vos A. De Munck N. (2025). Trophectoderm biopsy: present state of the art. Genes 16, 134–16. 10.3390/GENES16020134 40004463 PMC11854799

[B47] De Wert G. Mummery C. (2003). Human embryonic stem cells: research, ethics and policy. Hum. Reprod. 18, 672–682. 10.1093/HUMREP/DEG143 12660256

[B48] Delhove J. Osenk I. Prichard I. Donnelley M. (2020). Public acceptability of gene therapy and gene editing for human use: a systematic review. Hum. Gene Ther. 31, 20–46. 10.1089/hum.2019.197 31802714

[B49] Di Bello F. Creta M. Napolitano L. Califano G. Passaro F. Morra S. (2022). Male sexual dysfunction and infertility in spinal cord injury patients: state-Of-The-art and future perspectives. J. Pers. Med. 12, 873. 10.3390/JPM12060873 35743658 PMC9225464

[B50] Diaz P. Dullea A. Chu K. Y. Zizzo J. Loloi J. Reddy R. (2022). Future of Male infertility evaluation and treatment: brief review of emerging technology. Urology 169, 9–16. 10.1016/J.UROLOGY.2022.06.036 35905774

[B51] Ding X. Schimenti J. C. (2023). Female infertility from oocyte maturation arrest: assembling the genetic puzzle. EMBO Mol. Med. 15, e17729. 10.15252/EMMM.202317729 37073822 PMC10245026

[B52] Dissanayake D. M. I. H. Keerthirathna W. L. R. Peiris L. D. C. (2019). Male infertility problem: a contemporary review on present status and future perspective. Gend. Genome 3, 247028971986824. 10.1177/2470289719868240

[B53] Donnez J. Dolmans M. Demylle D. Jadoul P. Pirard C. Squifflet J. (2004). Livebirth after orthotopic transplantation of cryopreserved ovarian tissue. Lancet 364, 1405–1410. 10.1016/S0140-6736(04)17222-X 15488215

[B54] Drakopoulos P. Garcia-Velasco J. Bosch E. Blockeel C. de Vos M. Santos-Ribeiro S. (2019). ICSI does not offer any benefit over conventional IVF across different ovarian response categories in non-male factor infertility: a European multicenter analysis. J. Assist. Reprod. Genet. 36, 2067–2076. 10.1007/S10815-019-01563-1 31440957 PMC6823343

[B55] Duffy J. M. N. Adamson G. D. Benson E. Bhattacharya S. Bofill M. Brian K. (2021). Top 10 priorities for future infertility research: an international consensus development study. Fertil. Steril. 115, 180–190. 10.1016/J.FERTNSTERT.2020.11.014 33272617

[B56] Duguet A.-M. Rial-Sebbag E. Mahalatchimy A. Li M. Cambon-Thomsen A. (2018). Ethical and legal frameworks for embryonic stem-cell based research in France and in Europe: a challenge for biotechnology. HAL SHS Sci. humaines sociales.

[B57] Edwards R. G. Steptoe P. C. Purdy J. M. (1980). Establishing full-term human pregnancies using cleaving embryos grown *in vitro* . BJOG 87, 737–756. 10.1111/J.1471-0528.1980.TB04610.X 6775685

[B58] Edwards R. G. Purdy J. M. Steptoe P. C. Walters D. E. (1981). The growth of human preimplantation embryos *in vitro* . Am. J. Obstet. Gynecol. 141, 408–416. 10.1016/0002-9378(81)90603-7 7282823

[B59] Eisenberg M. L. Esteves S. C. Lamb D. J. Hotaling J. M. Giwercman A. Hwang K. (2023). Male infertility. Nat. Rev. Dis. Prim. 9, 49. 10.1038/s41572-023-00459-w 37709866

[B60] El Hachem H. Poulain M. Finet A. Fanchin R. Frydman N. Grynberg M. H. (2014). Live birth after frozen-thawed oocytes matured *in vitro* in a PCOS patient: a model for improving implantation rates in IVM cycles and objectively assessing the real potential of development of frozen oocytes matured *in vitro* . Gynecoll Endocrinol. 30, 415–418. 10.3109/09513590.2014.893573 24576224

[B61] El Hajj N. Haertle L. Dittrich M. Denk S. Lehnen H. Hahn T. (2017). DNA methylation signatures in cord blood of ICSI children. Hum. Reprod. 32, 1761–1769. 10.1093/HUMREP/DEX209 28575352 PMC5850272

[B62] Elder K. (2001). *In vitro* fertilization. Preimplantation Genet. Diagn., 53–78. 10.1002/0470846615.CH5

[B63] Esteves S. C. Sánchez-Martín F. Sánchez-Martín P. Schneider D. T. Gosálvez J. (2015). Comparison of reproductive outcome in oligozoospermic men with high sperm DNA fragmentation undergoing intracytoplasmic sperm injection with ejaculated and testicular sperm. Fertil. Steril. 104, 1398–1405. 10.1016/j.fertnstert.2015.08.028 26428305

[B64] Esteves S. C. Roque M. Bedoschi G. Haahr T. Humaidan P. (2018). Intracytoplasmic sperm injection for Male infertility and consequences for offspring. Nat. Rev. Urol. 15, 535–562. 10.1038/s41585-018-0051-8 29967387

[B65] Estill M. S. Bolnick J. M. Waterland R. A. Bolnick A. D. Diamond M. P. Krawetz S. A. (2016). Assisted reproductive technology alters deoxyribonucleic acid methylation profiles in bloodspots of newborn infants. Fertil. Steril. 106 (3), 629–63910. 10.1016/J.FERTNSTERT.2016.05.006 27288894

[B66] European Commission, and European Group on Ethics in Science and New Technologies (2018). Statement on gene editing. Available online at: https://coilink.org/20.500.12592/v18cz3 (Accessed March 4, 2025).

[B67] Ferronato G. de A. Vit F. F. Silveira J. C. da (2024). 3D culture applied to reproduction in females: possibilities and perspectives. Anim. Reprod. 21, e20230039. 10.1590/1984-3143-ar2023-0039 38510565 PMC10954237

[B68] Fesahat F. Montazeri F. Hoseini S. M. (2020). Preimplantation genetic testing in assisted reproduction technology. J. Gynecol. Obstet. Hum. Reprod. 49, 101723. 10.1016/J.JOGOH.2020.101723 32113002

[B69] Filho E. S. Noble J. A. Poli M. Griffiths T. Emerson G. Wells D. (2012). A method for semi-automatic grading of human blastocyst microscope images. Hum. Reprod. 27, 2641–2648. 10.1093/HUMREP/DES219 22736327

[B70] Francés-Herrero E. Lopez R. Hellström M. de Miguel-Gómez L. Herraiz S. Brännström M. (2022). Bioengineering trends in female reproduction: a systematic review. Hum. Reprod. Update 28, 798–837. 10.1093/humupd/dmac025 35652272 PMC9629485

[B71] Fuchs I. (2024). Law vs. ethics: the debate over what’s legal and what’s right. American Public University. Available online at: https://www.apu.apus.edu/area-of-study/security-and-global-studies/resources/law-vs-ethics/?_x_tr_sl&_x_tr_tl&_x_tr_hl (Accessed March 5, 2025).

[B72] Funk C. Hefferon M. (2018). Public views of gene editing for babies depend on how it would be used. Available online at: https://www.pewresearch.org/science/2018/07/26/public-views-of-gene-editing-for-babies-depend-on-how-it-would-be-used/(Accessed March 4, 2025).

[B73] García-Pascual C. M. Navarro-Sánchez L. Navarro R. Martínez L. Jiménez J. Rodrigo L. (2020). Optimized ngs approach for detection of aneu-ploidies and mosaicism in pgt-a and imbalances in pgt-sr. Genes (Basel) 11, 1–10. 10.3390/genes11070724 32610655 PMC7397276

[B74] Garcia-Velasco J. A. Domingo J. Cobo A. Martínez M. Carmona L. Pellicer A. (2013). Five years’ experience using oocyte vitrification to preserve fertility for medical and nonmedical indications. Fertil. Steril. 99, 1994–1999. 10.1016/j.fertnstert.2013.02.004 23465707

[B75] Geng T. Cheng L. Ge C. Zhang Y. (2020). The effect of ICSI in infertility couples with non-male factor: a systematic review and meta-analysis. J. Assist. Reprod. Genet. 37, 2929–2945. 10.1007/S10815-020-01970-9 33073301 PMC7714870

[B76] Ghanbari E. Khazaei M. Ghahremani-nasab M. Mehdizadeh A. Yousefi M. (2020). Novel therapeutic approaches of tissue engineering in Male infertility. Cell Tissue Res. 380, 31–42. 10.1007/s00441-020-03178-w 32043209

[B77] Glina S. Vieira M. (2013). Prognostic factors for sperm retrieval in non-obstructive azoospermia. Clinics 68, 121–124. 10.6061/CLINICS/2013(SUP01)13 23503961 PMC3583147

[B78] Głowacka M. (2018). Problem niepłodności wśród kobiet i mężczyzn-epidemiologia, czynniki ryzyka i świadomość społeczna. Available online at: https://www.researchgate.net/publication/325012974.

[B79] Gontar J. Kazachkova N. Ilyin I. Buderatska N. Fedota A. Parnitska O. (2019). PGT-A, a new dance for two couples: NGS with FISH and trophectoderm cells with blastocelic fluid. Reprod. Biomed. Online 38, e42. 10.1016/j.rbmo.2019.03.069

[B80] Goodson S. G. White S. Stevans A. M. Bhat S. Kao C. Y. Jaworski S. (2017). CASAnova: a multiclass support vector machine model for the classification of human sperm motility patterns. Biol. Reprod. 97, 698–708. 10.1093/biolre/iox120 29036474 PMC6248632

[B81] Greco E. Litwicka K. Minasi M. G. Cursio E. Greco P. F. Barillari P. (2020). Preimplantation genetic testing: where we are today. Int. J. Mol. Sci. 21, 4381. 10.3390/IJMS21124381 32575575 PMC7352684

[B82] Grosicka-Maciag E. (2011). Biologiczne skutki stresu oksydacyjnego wywołanego działaniem pestycydów. Postepy Hig. Med. Dosw 65, 357–366. 10.5604/17322693.948816 21734320

[B83] Gudeloglu A. Brahmbhatt J. V. Parekattil S. J. (2014). Medical management of male infertility in the absence of a specific etiology. Semin. Reprod. Med. 32, 313–318. 10.1055/s-0034-1375184 24919031

[B84] Gul M. Russo G. I. Kandil H. Boitrelle F. Saleh R. Chung E. (2024). Male infertility: new developments, current challenges, and future directions. World J. Mens. Health 42, 502–517. 10.5534/WJMH.230232 38164030 PMC11216957

[B85] Guo X. Y. Liu X. M. Jin L. Wang T. T. Ullah K. Sheng J. Z. (2017). Cardiovascular and metabolic profiles of offspring conceived by assisted reproductive technologies: a systematic review and meta-analysis. Fertil. Steril. 107, 622–631.e5. 10.1016/J.FERTNSTERT.2016.12.007 28104241

[B86] Guo Y. Dong Y. Zheng R. Yan J. Li W. Xu Y. (2024). Correlation between viral infections in Male semen and infertility: a literature review. Virol. J. 21, 167–19. 10.1186/S12985-024-02431-W 39080728 PMC11290048

[B87] Haddad M. Stewart J. Xie P. Cheung S. Trout A. Keating D. (2021). Thoughts on the popularity of ICSI. J. Assist. Reprod. Genet. 38, 101–123. 10.1007/S10815-020-01987-0 33155089 PMC7823003

[B88] Haider S. Beristain A. G. (2023). Human organoid systems in modeling reproductive tissue development, function, and disease. Hum. Reprod. 38, 1449–1463. 10.1093/humrep/dead085 37119533

[B89] Hamberger L. Lundin K. Sjögren A. Söderlund B. (1998). Indications for intracytoplasmic sperm injection. Hum. Reprod. 13, 128–133. 10.1093/HUMREP/13.SUPPL_1.128 9663777

[B90] Handyside A. H. Kontogianni E. H. Hardy K. Winston R. M. L. (1990). Pregnancies from biopsied human preimplantation embryos sexed by Y-specific DNA amplification. Nature 344, 768–770. 10.1038/344768a0 2330030

[B91] Handyside A. H. Lesko J. G. Tarín J. J. Winston R. M. Hughes M. R. (1992). Birth of a normal girl after *in vitro* fertilization and preimplantation diagnostic testing for cystic fibrosis. N. Engl. J. Med. 327, 905–909. 10.1056/NEJM199209243271301 1381054

[B92] Hanson B. Johnstone E. Dorais J. Silver B. Peterson C. M. Hotaling J. (2017). Female infertility, infertility-associated diagnoses, and comorbidities: a review. J. Assist. Reprod. Genet. 34, 167–177. 10.1007/S10815-016-0836-8 27817040 PMC5306404

[B93] Hariton E. Alvero R. Hill M. J. Mersereau J. E. Perman S. Sable D. (2023). Meeting the demand for fertility services: the present and future of reproductive endocrinology and infertility in the United States. Fertil. Steril. 120, 755–766. 10.1016/J.FERTNSTERT.2023.08.019 37665313

[B94] Harris J. (2010). Enhancing evolution: the ethical case for making better people. Princeton University Press. Available online at: http://digital.casalini.it/9781400836383 (Accessed March 23, 2025).

[B95] Heape W. (1891). Preliminary note on the transplantation and growth of Mammalian ova within a uterine foster-mother. Proc. R. Soc. Lond. 48, 457–458. 10.1098/RSPL.1890.0053

[B96] Heape W. Marshall F. H. A. Betteridge K. J. (1981). An historical look at embryo transfer. Reproduction 62, 1–13. 10.1530/JRF.0.0620001 7014855

[B97] Heidari-Khoei H. Esfandiari F. Hajari M. A. Ghorbaninejad Z. Piryaei A. Piryaei A. (2020). Organoid technology in female reproductive biomedicine. Reprod. Biol. Endocrinol. 18, 64. 10.1186/s12958-020-00621-z 32552764 PMC7301968

[B98] Henkel R. R. (2010). Leukocytes and oxidative stress: dilemma for sperm function and Male fertility. Asian J. Androl. 13, 43–52. 10.1038/AJA.2010.76 21076433 PMC3739401

[B99] Hofmann B. (2025). Bioethics: no method—no discipline? Cam Q. Healthc. Ethics 34, 99–108. 10.1017/S0963180124000136 38515428

[B100] Hou W. Shi G. Ma Y. Liu Y. Lu M. Fan X. (2021). Impact of preimplantation genetic testing on obstetric and neonatal outcomes: a systematic review and meta-analysis. Fertil. Steril. 116, 990–1000. 10.1016/j.fertnstert.2021.06.040 34373103

[B101] Houston B. J. Riera-Escamilla A. Wyrwoll M. J. Salas-Huetos A. Xavier M. J. Nagirnaja L. (2021). A systematic review of the validated monogenic causes of human Male infertility: 2020 update and a discussion of emerging gene–disease relationships. Hum. Reprod. Update 28, 15–29. 10.1093/HUMUPD/DMAB030 34498060 PMC8730311

[B102] Iqbal M. J. Javed Z. Sadia H. Qureshi I. A. Irshad A. Ahmed R. (2021). Clinical applications of artificial intelligence and machine learning in cancer diagnosis: looking into the future. Cancer Cell Int. 21, 270. 10.1186/s12935-021-01981-1 34020642 PMC8139146

[B103] Jamalirad H. Jajroudi M. Khajehpour B. Sadighi Gilani M. A. Eslami S. Sabbaghian M. (2025). AI predictive models and advancements in microdissection testicular sperm extraction for non-obstructive azoospermia: a systematic scoping review. Hum. Reprod. Open 2025, hoae070. 10.1093/hropen/hoae070 39764557 PMC11700607

[B104] Jensen C. F. S. Dong L. Gul M. Fode M. Hildorf S. Thorup J. (2022). Fertility preservation in boys facing gonadotoxic cancer therapy. Nat. Rev. Urol. 19, 71–83. 10.1038/s41585-021-00523-8 34667304

[B105] Jiang V. S. Bormann C. L. (2023). Artificial intelligence in the *in vitro* fertilization laboratory: a review of advancements over the last decade. Fertil. Steril. 120, 17–23. 10.1016/j.fertnstert.2023.05.149 37211062

[B106] Joris H. De Vos A. Janssens R. Devroey P. Liebaers I. Van Steirteghem A. (2003). Comparison of the results of human embryo biopsy and outcome of PGD after zona drilling using acid tyrode medium or a laser. Hum. Reprod. 18, 1896–1902. 10.1093/HUMREP/DEG355 12923146

[B107] Juengst E. Fossel M. (2000). The ethics of embryonic stem cells—now and forever, cells without end. JAMA 284, 3180–3184. 10.1001/JAMA.284.24.3180 11135785

[B108] Kamel R. M. (2013). Assisted reproductive technology after the birth of louise brown. J. Reprod. Infertil. 14, 96–109. 10.4172/2161-0932.1000156 24163793 PMC3799275

[B109] Kanakasabapathy M. K. Thirumalaraju P. Bormann C. L. Gupta R. Pooniwala R. Kandula H. (2020). Deep learning mediated single time-point image-based prediction of embryo developmental outcome at the cleavage stage. arXiv., 8346. 10.48550/arXiv.2006.08346

[B110] Khan F. A. Pandupuspitasari N. S. ChunJie H. Ahmad H. I. Wang K. Ahmad M. J. (2018). Applications of CRISPR/Cas9 in reproductive biology. Curr. Issues Mol. Biol. 26, 93–102. 10.21775/cimb.026.093 28879859

[B111] Kohli M. Prevedello L. M. Filice R. W. Geis J. R. (2017). Implementing machine learning in radiology practice and research. AMR Am. J. Roentgenol. 208, 754–760. 10.2214/AJR.16.17224 28125274

[B112] Kuliev A. Rechitsky S. (2011). Polar body-based preimplantation genetic diagnosis for Mendelian disorders. Mol. Hum. Reprod. 17, 275–285. 10.1093/MOLEHR/GAR012 21320873

[B113] Kumar N. Singh A. (2015). Trends of Male factor infertility, an important cause of infertility: a review of literature. J. Hum. Reprod. Sci. 8, 191–196. 10.4103/0974-1208.170370 26752853 PMC4691969

[B114] Kumar N. Singh A. K. (2022). Impact of environmental factors on human semen quality and Male fertility: a narrative review. Environ. Sci. Eur. 34, 6–13. 10.1186/S12302-021-00585-W

[B115] Kwack S. J. Lee B. M. (2015). Comparative cytotoxicity and sperm motility using a computer-aided sperm analysis system (CASA) for isomers of phthalic acid, a common final metabolite of phthalates. J. Toxicol. Environ. Health A 78, 1038–1050. 10.1080/15287394.2015.1067503 26252616

[B116] Łakoma K. Kukharuk O. Śliż D. (2023). The influence of metabolic factors and diet on fertility. Nutrients 15, 1180. 10.3390/NU15051180 36904180 PMC10005661

[B117] Lamb D. J. Niederberger C. S. (1993). Artificial intelligence in medicine and Male infertility. World J. Urol. 11, 129–136. 10.1007/BF00182040 8343796

[B118] Lancaster M. A. Knoblich J. A. (2014). Organogenesis in a dish: modeling development and disease using organoid technologies. Science 345, 1247125. 10.1126/SCIENCE.1247125 25035496

[B119] Laronda M. M. (2020). Engineering a bioprosthetic ovary for fertility and hormone restoration. Theriogenology 150, 8–14. 10.1016/j.theriogenology.2020.01.021 31973967

[B120] Lasota J. (2023). Porównanie metod sztucznej inteligencji stosowanych w diagnostyce preimplantacyjnej przy zapłodnieniu pozaustrojowym. Available online at: https://ruj.uj.edu.pl/xmlui/handle/item/312917 (Accessed April 18, 2025).

[B121] Lee B. Lee K. Panda S. Gonzales-Rojas R. Chong A. Bugay V. (2018). Nanoparticle delivery of CRISPR into the brain rescues a mouse model of fragile X syndrome from exaggerated repetitive behaviours. Nat. Biomed. Eng. 2, 497–507. 10.1038/s41551-018-0252-8 30948824 PMC6544395

[B122] Lewis S. E. M. Agbaje I. Alvarez J. (2008). Sperm DNA tests as useful adjuncts to semen analysis. Syst. Biol. Reprod. Med. 54, 111–125. 10.1080/19396360801957739 18570047

[B123] Li Y. Pan P. Yuan P. Qiu Q. Yang D. (2016). Successful live birth in a woman with resistant ovary syndrome following *in vitro* maturation of oocytes. J. Ovarian Res. 9, 54. 10.1186/s13048-016-0263-6 27599836 PMC5012097

[B124] Li Z. Wang A. Y. Bowman M. Hammarberg K. Farquhar C. Johnson L. (2018). ICSI does not increase the cumulative live birth rate in non-male factor infertility. Hum. Reprod. 33, 1322–1330. 10.1093/humrep/dey118 29897449

[B125] Liang P. Xu Y. Zhang X. Ding C. Huang R. Zhang Z. (2015). CRISPR/Cas9-mediated gene editing in human tripronuclear zygotes. Protein Cell 6, 363–372. 10.1007/s13238-015-0153-5 25894090 PMC4417674

[B126] Liu K. Zhang Y. Martin C. Ma X. Shen B. (2022). Translational bioinformatics for human reproductive biology research: examples, opportunities and challenges for a future reproductive medicine. Int. J. Mol. Sci. 24, 4. 10.3390/IJMS24010004 36613446 PMC9819745

[B127] Liu X. Zhang Q. Cao K. Li J. Gao Y. Xu P. (2025). Preimplantation genetic testing for monogenic disorders (PGT-M) for monogenic nephropathy: a single-center retrospective cohort analysis. Clin. Kidney J. 18, sfae356. 10.1093/ckj/sfae356 39802589 PMC11719030

[B128] Long S. Fu L. Ma J. Yu H. Tang X. Hu T. (2024). Novel biallelic variants in *DNAH1* cause multiple morphological abnormalities of sperm flagella with favorable outcomes of fertility after ICSI in han Chinese males. Andrology 12, 349–364. 10.1111/andr.13476 37302001

[B129] Lustgarten Guahmich N. Borini E. Zaninovic N. (2023). Improving outcomes of assisted reproductive technologies using artificial intelligence for sperm selection. Fertil. Steril. 120, 729–734. 10.1016/j.fertnstert.2023.06.009 37307892

[B130] Ma Y. Gu M. Chen L. Shen H. Pan Y. Pang Y. (2021). Recent advances in critical nodes of embryo engineering technology. Theranostics 11, 7391–7424. 10.7150/THNO.58799 34158857 PMC8210615

[B131] Madjunkova S. Agrawal A. Chen S. Abramov R. Logan N. Glass K. (2024). P-551 Application of simultaneous PGT-M/PGT-A using whole genome sequencing and haplotyping for monogenetic disorders, multiple variants, low penetrance, and *de novo* disorders. Hum. Reprod. 39, deae108.200. 10.1093/HUMREP/DEAE108.200

[B132] Makar R. S. Toth T. L. (2002). The evaluation of infertility. Pathol. Patterns Rev. 117, S95–S103. 10.1309/W8LJ-K377-DHRA-CP0B 14569805

[B133] Makrakis E. Angeli I. Agapitou K. Pappas K. Dafereras A. Pantos K. (2006). Laser *versus* mechanical assisted hatching: a prospective study of clinical outcomes. Fertil. Steril. 86, 1596–1600. 10.1016/j.fertnstert.2006.05.031 17055494

[B134] Mamas T. Kakourou G. Vrettou C. Traeger-Synodinos J. (2022). Hemoglobinopathies and preimplantation diagnostics. Int. J. Lab. Hematol. 44, 21–27. 10.1111/IJLH.13851 35443077

[B135] Mandai M. Watanabe A. Kurimoto Y. Hirami Y. Morinaga C. Daimon T. (2017). Autologous induced stem-Cell–Derived retinal cells for macular degeneration. N. Engl. J. Med. 376, 1038–1046. 10.1056/NEJMoa1608368 28296613

[B136] Mao D. Xu J. Sun L. (2024). Impact of trophectoderm biopsy for preimplantation genetic testing on obstetric and neonatal outcomes: a meta-analysis. Am. J. Obstet. Gynecol. 230, 199–212.e5. 10.1016/J.AJOG.2023.08.010 37595823

[B137] Martino E.De Kopcow L. Papayannis M. Zúñiga I. de Horton M. Oubiña A. (2024). P-221 the efficiency of a PGT-A program is negatively impacted by the utilization of frozen-thawed oocytes in patients of all ages. Hum. Reprod. 39, deae108.591. 10.1093/HUMREP/DEAE108.591

[B138] Massarotti C. Sbragia E. Gazzo I. Stigliani S. Inglese M. Anserini P. (2021). Effect of multiple sclerosis and its treatments on Male fertility: Cues for future research. J. Clin. Med. 10, 5401. 10.3390/JCM10225401 34830684 PMC8623707

[B139] Matthews K. R. W. Morali D. (2022). Can we do that here? An analysis of US federal and state policies guiding human embryo and embryoid research. J. Law Biosci. 9, lsac014. 10.1093/jlb/lsac014 35692936 PMC9183789

[B140] McCaughey T. Sanfilippo P. G. Gooden G. E. C. Budden D. M. Fan L. Fenwick E. (2016). A global social media survey of attitudes to human genome editing. Cell Stem Cell 18, 569–572. 10.1016/j.stem.2016.04.011 27152441

[B141] Medenica S. Zivanovic D. Batkoska L. Marinelli S. Basile G. Perino A. (2022). The future is coming: artificial intelligence in the treatment of infertility could improve assisted reproduction outcomes—the value of regulatory frameworks. Diagnostics 12, 2979. 10.3390/DIAGNOSTICS12122979 36552986 PMC9777042

[B142] Mehravar M. Shirazi A. Nazari M. Banan M. (2019). Mosaicism in CRISPR/Cas9-mediated genome editing. Dev. Biol. 445, 156–162. 10.1016/j.ydbio.2018.10.008 30359560

[B143] Menkin M. F. Rock J. (1948). *In vitro* fertilization and cleavage of human ovarian eggs. Am. J. Obstet. Gynecol. 55, 440–452. 10.1016/S0002-9378(15)32963-X 18903892

[B144] Mohiuddin M. Kooy R. F. Pearson C. E. (2022). *De novo* mutations, genetic mosaicism and human disease. Front. Genet. 13, 983668. 10.3389/fgene.2022.983668 36226191 PMC9550265

[B145] Moustakli E. Gkountis A. Dafopoulos S. Zikopoulos A. Sotiriou S. Zachariou A. (2024). Comparative analysis of fluorescence *in situ* hybridization and next-generation sequencing in sperm evaluation: implications for preimplantation genetic testing and Male infertility. Inte J. Mol. Sci. 25, 11296. 10.3390/IJMS252011296 39457078 PMC11508275

[B146] Mulvihill J. J. Capps B. Joly Y. Lysaght T. Zwart H. A. E. Chadwick R. (2017). Ethical issues of CRISPR technology and gene editing through the lens of solidarity. Br. Med. Bull. 122, 17–29. 10.1093/bmb/ldx002 28334154

[B147] Murakami K. Hamazaki N. Hamada N. Nagamatsu G. Okamoto I. Ohta H. (2023). Generation of functional oocytes from Male mice *in vitro* . Nature 615, 900–906. 10.1038/S41586-023-05834-X 36922585

[B148] Nair R. Kasturi M. Mathur V. Seetharam R. N. S Vasanthan K. (2024). Strategies for developing 3D printed ovarian model for restoring fertility. Clin. Transl. Sci. 17, e13863. 10.1111/cts.13863 38955776 PMC11219245

[B149] Netherton J. K. Hetherington L. Ogle R. A. Gavgani M. M. Velkov T. Villaverde A. I. B. (2020). Mass spectrometry reveals new insights into the production of superoxide anions and 4-Hydroxynonenal adducted proteins in human sperm. Proteomics 20, e1900205. 10.1002/PMIC.201900205 31846556

[B150] Ní Dhonnabháin B. Elfaki N. Fraser K. Petrie A. Jones B. P. Saso S. (2022). A comparison of fertility preservation outcomes in patients who froze oocytes, embryos, or ovarian tissue for medically indicated circumstances: a systematic review and meta-analysis. Fertil. Steril. 117, 1266–1276. 10.1016/j.fertnstert.2022.03.004 35459522

[B151] Nie J. Han Y. Jin Z. Hang W. Shu H. Wen Z. (2023). Homology-directed repair of an MYBPC3 gene mutation in a rat model of hypertrophic cardiomyopathy. Gene Ther. 30, 520–527. 10.1038/s41434-023-00384-3 36765144

[B152] Niederberger C. Pellicer A. Cohen J. Gardner D. K. Palermo G. D. O’Neill C. L. (2018). Forty years of IVF. Fertil. Steril. 110, 185–324.e5. 10.1016/J.FERTNSTERT.2018.06.005 30053940

[B153] Nikiforov D. Grøndahl M. L. Hreinsson J. Andersen C. Y. (2022). Human oocyte morphology and outcomes of infertility treatment: a systematic review. Reprod. Sci. 29, 2768–2785. 10.1007/S43032-021-00723-Y 34816375

[B154] Nishio E. Moriwaki T. Yoshii K. Udagawa Y. (2006). Chemical removal of zona pellucida *versus* laser assisted hatching after repeated failures of assisted reproductive technology. Reprod. Med. Biol. 5, 263–267. 10.1111/J.1447-0578.2006.00151.X 29699255 PMC5904657

[B155] Nnaji N. D. Onyeaka H. Miri T. Ugwa C. (2023). Bioaccumulation for heavy metal removal: a review. SN Appl. Sci. 5, 125–12. 10.1007/S42452-023-05351-6

[B156] O’Flaherty C. (2025). Redox signaling regulation in human spermatozoa: a primary role of peroxiredoxins. Asian J. Androl. 10.4103/AJA2024126 PMC12422578 39902615

[B243] Oktay K. Buyuk E. Davis O. Yermakova I. Veeck L. Rosenwaks Z. (2003). Fertility preservation in breast cancer patients: IVF and embryo cryopreservation after ovarian stimulation with tamoxifen. Hum. Reprod. 18, 90–95. 10.1093/humrep/deg045 12525446

[B157] Pagliardini L. Viganò P. Alteri A. Corti L. Somigliana E. Papaleo E. (2020). Shooting STAR: reinterpreting the data from the ‘Single Embryo TrAnsfeR of Euploid Embryo’ randomized clinical trial. Reprod. Biomed. Online 40, 475–478. 10.1016/j.rbmo.2020.01.015 32273162

[B158] Palermo G. Joris H. Devroey P. Van Steirteghem A. C. (1992). Pregnancies after intracytoplasmic injection of single spermatozoon into an oocyte. Lancet 340, 17–18. 10.1016/0140-6736(92)92425-F 1351601

[B244] Pandey S. Shetty A. Hamilton M. Bhattacharya S. Maheshwari A. (2012). Obstetric and perinatal outcomes in singleton pregnancies resulting from IVF/ICSI: a systematic review and meta-analysis. Hum. Reprod. Update 18, 485–503. 10.1093/humupd/dms018 22611174

[B159] Panner Selvam M. K. Moharana A. K. Baskaran S. Finelli R. Hudnall M. C. Sikka S. C. (2024). Current updates on involvement of artificial intelligence and machine learning in semen analysis. Med. Lith. 60, 279. 10.3390/medicina60020279 38399566 PMC10890589

[B160] Park J. Y. Lim H. Qin J. Lee L. P. (2023). Creating mini-pregnancy models *in vitro* with clinical perspectives. EBioMedicine 95, 104780. 10.1016/J.EBIOM.2023.104780 37657136 PMC10480532

[B161] Passet-Wittig J. Greil A. L. (2021). Factors associated with medical help-seeking for infertility in developed countries: a narrative review of recent literature. Soc. Sci. Med. 277, 113782. 10.1016/J.SOCSCIMED.2021.113782 33895708

[B162] Patrício D. Santiago J. Mano J. F. Fardilha M. (2023). Organoids of the Male reproductive system: challenges, opportunities, and their potential use in fertility research. WIREs Mech. Dis. 15, e1590. 10.1002/wsbm.1590 36442887

[B163] Peng Y.-J. Huang X. Zhou Q. (2020). Ethical and policy considerations for human embryo and stem cell research in China. Cell Stem Cell 27, 511–514. 10.1016/j.stem.2020.09.010 33007234

[B164] Piyamongkol W. (2020). Pre-implantation genetic testing for aneuploidy (PGT-A). Thai J. Obstet. Gynaecol. 28, 130–135. 10.14456/tjog.2020.17

[B165] Practice Committee of the American Society for Reproductive Medicine (2019). Fertility preservation in patients undergoing gonadotoxic therapy or gonadectomy: a committee opinion. Fertil. Steril. 112, 1022–1033. 10.1016/j.fertnstert.2019.09.013 31843073

[B166] Prokhorovich M. Rechitsky S. Pakhalchuk T. Ramon G. S. Gershman R. Bond E. (2019). 40. Simultaneous preimplantation genetic testing (Pgt) for 5 different genetic conditions. Reprod. Biomed. Online 39, e51. 10.1016/j.rbmo.2019.04.093

[B167] Pryzhkova M. V. Boers R. Jordan P. W. (2022). Modeling human gonad development in organoids. Tissue Eng. Regen. Med. 19, 1185–1206. 10.1007/S13770-022-00492-Y 36350469 PMC9679106

[B168] Qi L. Liu Y. P. Zhang N. N. Su Y. C. (2021). Predictors of testicular sperm retrieval in patients with non-obstructive azoospermia: a review. J. Int. Med. Res. 49, 3000605211002703. 10.1177/03000605211002703 33794677 PMC8020245

[B169] Rahbaran M. Razeghian E. Maashi M. S. Jalil A. T. Widjaja G. Thangavelu L. (2021). Cloning and embryo splitting in mammalians: brief history, methods, and achievements. Stem Cells Int. 2021, 2347506. 10.1155/2021/2347506 34887927 PMC8651392

[B170] Raposo V. L. (2019). The first Chinese edited babies: a leap of faith in science. JBRA Assist. Reprod. 23, 197–199. 10.5935/1518-0557.20190042 31436399 PMC6724388

[B171] Raposo V. L. Ma Z. (2020). An ethical evaluation of the legal status of foetuses and embryos under Chinese law. Dev. World Bioeth. 20, 38–49. 10.1111/dewb.12241 31359558

[B172] Richer G. Hobbs R. M. Loveland K. L. Goossens E. Baert Y. (2021). Long-term maintenance and meiotic entry of early germ cells in murine testicular organoids functionalized by 3D printed scaffolds and air-medium interface cultivation. Front. Physiol. 12, 757565. 10.3389/fphys.2021.757565 35002756 PMC8739976

[B173] Rooney K. L. Domar A. D. (2018). The relationship between stress and infertility. Dialogues Clin. Neurosci. 20, 41–47. 10.31887/DCNS.2018.20.1/KLROONEY 29946210 PMC6016043

[B174] Ryu D. Y. Kim K. U. Kwon W. S. Rahman M. S. Khatun A. Pang M. G. (2017). Peroxiredoxin activity is a major landmark of Male fertility. Sci. Rep. 7, 17174–13. 10.1038/s41598-017-17488-7 29215052 PMC5719347

[B175] Saeedi P. Yee D. Au J. Havelock J. (2017). Automatic identification of human blastocyst components *via* texture. IEEE Trans. Biomed. Eng. 64, 2968–2978. 10.1109/TBME.2017.2759665 28991729

[B176] Salas-Huetos A. Aston K. I. (2021). Defining new genetic etiologies of Male infertility: progress and future prospects. Transl. Androl. Urol. 10, 1486–1498. 10.21037/TAU.2020.03.43 33850783 PMC8039605

[B177] Salmeri N. Alteri A. Farina A. Pozzoni M. Vigano’ P. Candiani M. (2024). Preterm birth in singleton pregnancies conceived by *in-vitro* fertilization or intracytoplasmic sperm injection: an overview of systematic reviews. Am. J. Obstet. Gynecol. 231, 501–515.e9. 10.1016/J.AJOG.2024.05.037 38796038

[B178] Salvaggio C. N. Forman E. J. Garnsey H. M. Treff N. R. Scott R. T. (2014). Polar body based aneuploidy screening is poorly predictive of embryo ploidy and reproductive potential. J. Assist. Reprod. Genet. 31, 1221–1226. 10.1007/S10815-014-0293-1 25106935 PMC4156943

[B179] Sang Q. Ray P. F. Wang L. (2023). Understanding the genetics of human infertility. Science 380, 158–163. 10.1126/SCIENCE.ADF7760 37053320

[B180] Santoro M. De Amicis F. Aquila S. Bonofiglio D. (2020). Peroxisome proliferator-activated receptor gamma expression along the Male genital system and its role in Male fertility. Hum. Reprod. 35, 2072–2085. 10.1093/humrep/deaa153 32766764

[B181] Schlegel P. N. Sigman M. Collura B. De Jonge C. J. Eisenberg M. L. Lamb D. J. (2021). Diagnosis and treatment of infertility in men: AUA/ASRM guideline part I. J. Urol. 205, 36–43. 10.1097/JU.0000000000001521 33295257

[B182] Sciorio R. Esteves S. C. (2022). Contemporary use of ICSI and epigenetic risks to future generations. J. Clin. Med. 11, 2135. 10.3390/JCM11082135 35456226 PMC9031244

[B183] Sciorio R. Tramontano L. Greco P. F. Greco E. (2024). Morphological assessment of oocyte quality during assisted reproductive technology cycle. JBRA Assist. Reprod. 28, 511–520. 10.5935/1518-0557.20240034 38801314 PMC11349268

[B184] Sekhon L. Feuerstein J. L. Lee J. A. Flisser E. Copperman A. B. Grunfeld L. (2017). A comparison of preimplantation genetic testing (PGT) platforms: targeted next generation sequencing (NGS) results in comparable clinical outcomes despite identifying fewer suitable embryos for transfer. Fertil. Steril. 108, e269–e270. 10.1016/j.fertnstert.2017.07.803

[B185] Septiningrum L. Dewanti P. Aini Q. (2022). Male fertility classification based on life-style factors using logistic regression and support vector machine. AIP Conf. Proc. 2566, 030007. 10.1063/5.0114150

[B186] Sharma R. S. Saxena R. Singh R. (2018). Infertility and assisted reproduction: a historical and modern scientific perspective. Ind. J. Med. Res. 148, 10–14. 10.4103/IJMR.IJMR_636_18 30964077 PMC6469376

[B187] Sharma P. Malik S. Sarkar A. (2024). Exploring the idea of human reproduction in space: a potential area for future research. Cureus 6, e73712. 10.7759/cureus.73712 39677198 PMC11646162

[B188] Shetty S. Nair J. Johnson J. Shetty N. Kumar A. J. Thondehalmath N. (2022). Preimplantation genetic testing for couples with balanced chromosomal rearrangements. J. Reprod. Infertil. 23, 213–223. 10.18502/JRI.V23I3.10013 36415497 PMC9666592

[B189] Si K. Huang B. Jin L. (2023). Application of artificial intelligence in gametes and embryos selection. Hum. Fertil. 26, 757–777. 10.1080/14647273.2023.2256980 37705466

[B190] Simopoulou M. Sfakianoudis K. Maziotis E. Tsioulou P. Grigoriadis S. Rapani A. (2021). PGT-A: who and when? Α systematic review and network meta-analysis of RCTs. J. Assist. Reprod. Genet. 38, 1939–1957. 10.1007/S10815-021-02227-9 34036455 PMC8417193

[B191] Simpson J. L. Lamb D. J. (2001). Genetic effects of intracytoplasmic sperm injection. Semin. Reprod. Med. 19 (3), 239–249. 10.1055/S-2001-18043 11679905

[B192] Skakkebæk N. E. Lindahl-Jacobsen R. Levine H. Andersson A. M. Jørgensen N. Main K. M. (2021). Environmental factors in declining human fertility. Nat. Rev. Endocrinol. 18, 139–157. 10.1038/s41574-021-00598-8 34912078

[B193] Smeenk J. Wyns C. De Geyter C. Kupka M. Bergh C. Saiz I. C. (2023). ART in Europe, 2019: results generated from European registries by ESHRE. Hum. Reprod. 38, 2321–2338. 10.1093/HUMREP/DEAD197 37847771 PMC10694409

[B194] Steptoe P. Edwards R. G. (1978). Birth after the reim-plantation of a human embryo. Lancet 2, 366. 10.1016/s0140-6736(78)92957-4 79723

[B195] Suriyakalaa U. Ramachandran R. Doulathunnisa J. A. Aseervatham S. B. Sankarganesh D. Kamalakkannan S. (2021). Upregulation of Cyp19a1 and PPAR-γ in ovarian steroidogenic pathway by ficus religiosa: a potential cure for polycystic ovary syndrome. J. Ethnopharmacol. 267, 113540. 10.1016/j.jep.2020.113540 33152430

[B196] Surrey E. S. (2021). Mosaic embryo transfer: resolution or more controversy? Fertil. Steril. 115, 1160–1161. 10.1016/j.fertnstert.2021.02.044 33745727

[B197] Tafuri S. Ciani F. Iorio E. L. Cocchia L. E. and N. Tafuri S. Ciani F. (2015). “Reactive oxygen species (ROS) and Male fertility,” in New discoveries in embryology. 10.5772/60632

[B198] Takahashi K. Yamanaka S. (2006). Induction of pluripotent stem cells from mouse embryonic and adult fibroblast cultures by defined factors. Cell 126, 663–676. 10.1016/J.CELL.2006.07.024 16904174

[B199] Takahashi K. Tanabe K. Ohnuki M. Narita M. Ichisaka T. Tomoda K. (2007). Induction of pluripotent stem cells from adult human fibroblasts by defined factors. Cell 131, 861–872. 10.1016/j.cell.2007.11.019 18035408

[B200] Takeuchi K. (2021). Pre‐implantation genetic testing: past, present, future. Reprod. Med. Biol. 20, 27–40. 10.1002/RMB2.12352 33488281 PMC7812490

[B201] Tan V. J. Liu T. Arifin Z. Pak B. Tan A. S. C. Wong S. (2023). Third-generation single-molecule sequencing for preimplantation genetic testing of aneuploidy and segmental imbalances. Clin. Chem. 69, 881–889. 10.1093/clinchem/hvad062 37477572

[B202] Thomas J. Fishel S. B. Hall J. A. Green S. Newton T. A. Thornton S. J. (1997). Increased polymorphonuclear granulocytes in seminal plasma in relation to sperm morphology. Hum. Reprod. 12, 2418–2421. 10.1093/HUMREP/12.11.2418 9436676

[B203] Thomson J. A. Itskovitz-Eldor J. Shapiro S. S. Waknitz M. A. Swiergiel J. J. Marshall V. S. (1998). Embryonic stem cell lines derived from human blastocysts. Science 282, 1145–1147. 10.1126/SCIENCE.282.5391.1145 9804556

[B204] Tran K. T. D. Valli-Pulaski H. Colvin A. Orwig K. E. (2022). Male fertility preservation and restoration strategies for patients undergoing gonadotoxic therapies. Biol. Reprod. 107, 382–405. 10.1093/biolre/ioac072 35403667 PMC9382377

[B205] Urcelay L. Hinjos D. Martin-Torres P. A. Gonzalez M. Mendez M. Cívico S. (2023). “Exploring the role of explainability in AI-Assisted embryo selection,”Comput. Sci. - Comput. Vis. Pattern Recognit. 10.3233/FAIA230678

[B206] Valko M. Leibfritz D. Moncol J. Cronin M. T. D. Mazur M. Telser J. (2007). Free radicals and antioxidants in normal physiological functions and human disease. Int. J. Biochem. Cell Biol. 39, 44–84. 10.1016/J.BIOCEL.2006.07.001 16978905

[B207] Van Der Ven K. Montag M. Van Der Ven H. (2008). Polar body diagnosis – a step in the right direction? Dtsch. Arztebl Int. 105, 190–196. 10.3238/ARZTEBL.2008.0190 19629197 PMC2696746

[B208] van Marion E. S. Baart E. B. Santos M. van Duijn L. van Santbrink E. J. P. Steegers-Theunissen R. P. M. (2023). Using the embryo-uterus statistical model to predict pregnancy chances by using cleavage stage morphokinetics and female age: two centre-specific prediction models and mutual validation. Reprod. Biol. Endocrinol. 21, 31. 10.1186/S12958-023-01076-8 36973721 PMC10041771

[B209] Van Steirteghem A. Bonduelle M. Devroey P. Liebaers I. (2002). Follow-up of children born after ICSI. Hum. Reprod. Update 8 (2), 111–116. 10.1093/HUMUPD/8.2.111 12099626

[B210] Vander Borght M. Wyns C. (2018). Fertility and infertility: definition and epidemiology. Clin. Biochem. 62, 2–10. 10.1016/J.CLINBIOCHEM.2018.03.012 29555319

[B211] Vassena R. Heindryckx B. Peco R. Pennings G. Raya A. Sermon K. (2016). Genome engineering through CRISPR/Cas9 technology in the human germline and pluripotent stem cells. Hum. Reprod. Update 22, 411–419. 10.1093/humupd/dmw005 26932460

[B212] Venishetty N. Alkassis M. Raheem O. (2024). The role of artificial intelligence in Male infertility: evaluation and treatment: a narrative review. Uro 4, 23–35. 10.3390/URO4020003

[B213] Verpoest W. Staessen C. Bossuyt P. M. Goossens V. Altarescu G. Bonduelle M. (2018). Preimplantation genetic testing for aneuploidy by microarray analysis of polar bodies in advanced maternal age: a randomized clinical trial. Hum. Reprod. 33, 1767–1776. 10.1093/HUMREP/DEY262 30085138

[B214] Voelkerding K. V. Dames S. A. Durtschi J. D. (2009). Next-generation sequencing: from basic research to diagnostics. Clin. Chem. 55, 641–658. 10.1373/CLINCHEM.2008.112789 19246620

[B215] Vozdova M. Kubickova S. Rubes J. (2022). Spectrum of sperm mtDNA deletions in men exposed to industrial air pollution. Mutat. Res. Genet. Toxicol. Environ. Mutagen 882, 503538. 10.1016/J.MRGENTOX.2022.503538 36155140

[B216] Vuong L. N. Le A. H. Ho V. N. A. Pham T. D. Sanchez F. Romero S. (2020). Live births after oocyte *in vitro* maturation with a prematuration step in women with polycystic ovary syndrome. J. Assist. Reprod. Genet. 37, 347–357. 10.1007/s10815-019-01677-6 31902102 PMC7056678

[B217] Walczak-Jędrzejowska R. (2015). Oxidative stress and Male infertility. Part I: factors causing oxidative stress in semen. Post. Androl. Online 2 (1), 5–15.

[B218] Wang M. (2021). Next-generation sequencing (NGS). Clin. Mol. Diagn, 305–327. 10.1007/978-981-16-1037-0_23

[B219] Wang X. Wu H. He X. Jiang H. Wu L. Xu Y. (2018). Retrospective study to compare frozen-thawed embryo transfer with fresh embryo transfer on pregnancy outcome following intracytoplasmic sperm injection for Male infertility. Med. Sci. Monit. 24, 2668–2674. 10.12659/MSM.907229 29708103 PMC5946740

[B220] Wang R. Pan W. Jin L. Li Y. Geng Y. Gao C. (2019). Artificial intelligence in reproductive medicine. Reproduction 158, R139–R154. 10.1530/REP-18-0523 30970326 PMC6733338

[B221] Wang W. Guo J. Shi J. Li Q. Chen B. Pan Z. (2023). Bi-allelic pathogenic variants in PABPC1L cause oocyte maturation arrest and female infertility. EMBO Mol. Med. 15, e17177. 10.15252/EMMM.202217177 37052235 PMC10245037

[B222] Wang Y. Fu X. Li H. (2025). Mechanisms of oxidative stress-induced sperm dysfunction. Front. Endocrinol. 16, 1520835. 10.3389/fendo.2025.1520835 39974821 PMC11835670

[B223] Wdowiak N. Wójtowicz K. Wdowiak-Filip A. Pucek W. Wróbel A. Wróbel J. (2024). Environmental factors as the main hormonal disruptors of Male fertility. J. Clin. Med. 13, 1986. 10.3390/JCM13071986 38610751 PMC11012640

[B224] Wennerholm U. B. Bergh C. Hamberger L. Lundin K. Nlisson L. Wikland M. (2000). Incidence of congenital malformations in children born after ICSI. Hum. Reprod. 15 (4), 944–948. 10.1093/HUMREP/15.4.944 10739847

[B225] Wilson D. (2018). J. benjamin hurlbut. *Experiments in democracy: human embryo research and the politics of bioethics* . Isis 109 (2), 441–442. 10.1086/697919

[B226] Wu J. X. Xia T. She L. P. Lin S. Luo X. M. (2022). Stem cell therapies for human infertility: advantages and challenges. Cell Transpl. 31, 9636897221083252. 10.1177/09636897221083252 35348026 PMC8969497

[B227] Wyns C. De Geyter C. Calhaz-Jorge C. Kupka M. S. Motrenko T. Smeenk J. (2022). O-150 assisted reproductive technology (ART) in Europe 2019 and development of a strategy of vigilance preliminary results generated from European registers by the ESHRE EIM consortium. Hum. Reprod. 37, deac105.056. 10.1093/HUMREP/DEAC105.056

[B228] Xu X. Wang Z. Lv L. Liu C. Wang L. Sun Y. N. (2024). Molecular regulation of DNA damage and repair in female infertility: a systematic review. Reproductive Biol. Endocrinol. 22, 103–113. 10.1186/S12958-024-01273-Z 39143547 PMC11323701

[B229] Yahaya T. O. Liman U. U. Abdullahi H. Koko Y. S. Ribah S. S. Adamu Z. (2020). Genes predisposing to syndromic and nonsyndromic infertility: a narrative review. Egypt J. Med. Hum. Genet. 21, 46. 10.1186/s43042-020-00088-y

[B230] Yopo Díaz M. Watkins L. (2025). Beyond the body: social, structural, and environmental infertility. Soc. Sci. Med. 365, 117557. 10.1016/J.SOCSCIMED.2024.117557 39642584

[B231] Yu J. Thomson J. A. (2008). Pluripotent stem cell lines. Genes Dev. 22, 1987–1997. 10.1101/GAD.1689808 18676805 PMC2735345

[B232] Yuan S. Guo L. Cheng D. Li X. Hu H. Hu L. (2022). The *de novo* aberration rate of prenatal karyotype was comparable between 1496 fetuses conceived *via* IVF/ICSI and 1396 fetuses from natural conception. J. Assist. Reprod. Genet. 39, 1683–1689. 10.1007/S10815-022-02500-5 35616756 PMC9365907

[B233] Zaha I. Muresan M. Tulcan C. Huniadi A. Naghi P. Sandor M. (2023). The role of oxidative stress in infertility. J. Pers. Med. 13, 1264. 10.3390/JPM13081264 37623514 PMC10455473

[B234] Zarinara A. Zeraati H. Kamali K. Mohammad K. Shahnazari P. Akhondi M. M. (2016). Models predicting success of infertility treatment: a systematic review. J. Reprod. Infertil. 17, 68–81. 27141461 PMC4842237

[B235] Zeilmaker G. H. Alberda A. T. van Gent I. Rijkmans C. M. Drogendijk A. C. (1984). Two pregnancies following transfer of intact frozen-thawed embryos. Fertil. Steril. 42, 293–296. 10.1016/S0015-0282(16)48029-5 6745463

[B236] Zhai X. Ng V. Lie R. (2016). No ethical divide between China and the west in human embryo research. Dev. World Bioeth. 16, 116–120. 10.1111/dewb.12108 26791577

[B237] Zhang W. Y. Von Versen-Höynck F. Kapphahn K. I. Fleischmann R. R. Zhao Q. Baker V. L. (2019). Maternal and neonatal outcomes associated with trophectoderm biopsy. Obstet. Gynecol. Surv. 74, 657–658. 10.1097/01.OGX.0000612348.52166.23 PMC6527329 31103283

[B238] Zhang X. Yue Z. Zhang H. Liu L. Zhou X. (2020). Repeated administrations of Mn _3_ O _4_ nanoparticles cause testis damage and fertility decrease through PPAR-Signaling pathway. Nanotoxicology 14, 326–340. 10.1080/17435390.2019.1695976 31909642

[B239] Zheng Z. Chen L. Yang T. Yu H. Wang H. Qin J. (2018). Multiple pregnancies achieved with IVF/ICSI and risk of specific congenital malformations: a meta-analysis of cohort studies. Reprod. Biomed. Online 36 (4), 472–482. 10.1016/J.RBMO.2018.01.009 29609768

[B240] Zheng W. Yang C. Yang S. Sun S. Mu M. Rao M. (2021). Obstetric and neonatal outcomes of pregnancies resulting from preimplantation genetic testing: a systematic review and meta-analysis. Hum. Reprod. Update 27, 989–1012. 10.1093/HUMUPD/DMAB027 34473268

[B241] Zhou L. Liu H. Liu S. Yang X. Dong Y. Pan Y. (2023). Structures of sperm flagellar doublet microtubules expand the genetic spectrum of Male infertility. Cell 186, 2897–2910.e19. 10.1016/j.cell.2023.05.009 37295417

[B242] Zhu H. Shi L. Wang R. Cui L. Wang J. Tang M. (2022). Global research trends on infertility and psychology from the past two decades: a bibliometric and visualized study. Front. Endocrinol. 13, 889845. 10.3389/fendo.2022.889845 35903282 PMC9317298

